# mRNA Delivery by Lipoamino Fatty Acid–Peptide Polyplexes in Different Lung Cell Models and Lungs

**DOI:** 10.3390/polym18111368

**Published:** 2026-05-31

**Authors:** Sophie Thalmayr, Joschka Müller, Vivien Polewka, Irene Gialdini, Anny Nguyen, Christian Dohmen, Don C. Lamb, Olivia M. Merkel, Ernst Wagner

**Affiliations:** 1 Pharmaceutical Biotechnology, Department of Pharmacy, Ludwig-Maximilians-Universität München, 81377 Munich, Germany; sophie.thalmayr@cup.uni-muenchen.de; 2 Center for NanoScience (CeNS), Ludwig-Maximilians-Universität München, 80799 Munich, Germany; irene.gialdini@cup.lmu.de (I.G.); d.lamb@lmu.de (D.C.L.); olivia.merkel@lmu.de (O.M.M.); 3 CNATM—Cluster for Nucleic Acid Therapeutics Munich, 81377 Munich, Germany; joschka.mueller@cup.uni-muenchen.de (J.M.); polewka.vivien@mh-hannover.de (V.P.); anny.nguyen@cup.uni-muenchen.de (A.N.); dohmen@ethris.com (C.D.); 4 Pharmaceutical Technology and Biopharmaceutics, Department of Pharmacy, Ludwig-Maximilians-Universität München, 81377 Munich, Germany; 5 Ethris GmbH, 82152 Planegg, Germany; 6 Department of Chemistry, Ludwig-Maximilians-Universität München, 81377 Munich, Germany

**Keywords:** mRNA delivery, lungs, air–liquid interface, polyplex, shielding, hyaluronic acid, PEG, nanoparticles

## Abstract

Local pulmonary delivery offers a non-invasive application route for mRNA therapeutics with the potential for high bioavailability at the target-site of applications such as mucosal vaccination or the treatment of lung diseases. However, efficient delivery remains challenging due to major lung-specific barriers, particularly mucus. Herein, pH-responsive, amphiphilic xenopeptides comprising lipoamino fatty acids and oligoamino acids (OAAs) connected in distinct branched U-shape or bundle topologies were evaluated as mRNA polyplexes for delivery to A549 and Calu-3 lung cells under standard submerged or air–liquid interface (ALI) transfection conditions, and upon intratracheal application in BALB/c mice. Optionally, polyplexes were coated with negatively charged hyaluronic acid (HA) or colloidally stabilized with poly(ethylene glycol) (PEG). For U-shapes, hydrophobic modification of the OAA domain boosted their efficiency. Interestingly, best-performing formulations varied across transfection conditions. While the bundle topology showed the highest potential in submerged cell culture, U-shaped carriers were more efficient under ALI conditions. Polyplex surface modification with HA or PEG did not strongly alter in vitro transfections, whereas hydrophobized U-shape core polyplexes combined with surface modification enhanced their efficiency in vivo. Thus, the cationizable core and surface properties of mRNA nanoparticles require specific balancing in various lung cell models and lung.

## 1. Introduction

The approval and broad clinical application of mRNA vaccines [1,2,3] ushered in a new era for mRNA-based drugs with a pipeline of >400 therapies in clinical and preclinical development by the end of 2025 [4]. While a large research focus lies on invasive administration routes of mRNA drugs such as intramuscular or intravenous delivery, local topical administration to the lungs is another valuable drug delivery pathway. It represents a convenient, non-invasive administration route from the patient’s point of view. From the pharmacokinetic perspective, the lungs provide a large, strongly vascularized absorptive surface. Local administration circumvents the hepatic first-pass effect and enables bioavailability directly at the target site of lung diseases such as cystic fibrosis (CF) or chronic obstructive pulmonary disease without prior systemic passage [5,6]. Most recently, intratracheal delivery of cystic fibrosis transmembrane conductance regulator (CFTR) mRNA lipid nanoparticles (LNPs) restored the defective CFTR gene function in a ferret CF disease model [7]. Amongst other therapeutic strategies, currently three clinical trials for inhaled non-viral CFTR gene replacement using mRNA LNPs are ongoing [8]. Lung cancer or mucosal vaccination are further possible local pulmonary mRNA applications [9,10]. For example, five-component LNPs optimized with a negative zeta potential were successfully nebulized for inhalation, induced a robust mucosal and systemic immune responses against SARS-CoV-2 in mice and showed applicability for prophylactic and therapeutic cancer vaccination [10]. Polymer–lipid hybrid mRNA particles with a dual-targeting strategy of hyaluronic acid (HA) plus mannose effectively targeted and transfected lung cancer cells or proinflammatory macrophages in mouse models after inhalative administration [11]. Broad investigation of optimized ionizable lipids and formulation protocols paves the way for improved applicability and mRNA transfection efficiency of LNPs in local pulmonary delivery [12,13,14,15]. Polymeric- and polypeptide-based delivery systems are investigated as highly tunable alternative for non-viral local lung mRNA delivery even if not yet advanced to clinical stage [16,17,18,19,20].

These exemplary studies not only provide insight into the scientific progress of the field but also point to a critical aspect: pulmonary administration imposes unique requirements on nanoparticle drugs that differ from other administration routes. At first, they must cross lung fluids such as alveolar surfactant and bronchial mucus. The latter contains mucins, negatively charged glycoproteins with hydrophobic and hydrophilic domains as well as disulfide bonds that form a mesh structure, carbohydrates, lipids, salts, and free DNA and represents a substantial barrier for nanoparticles on their way towards the lung epithelium [5,6,21,22,23,24]. Two main clearance mechanisms represent additional delivery challenges. The mucociliary escalator continuously removes mucus from the respiratory tract to the esophagus, leading to a clearance within 15 min to 24 h. The pulmonary mononuclear phagocyte system can effectively clear nano- and microparticles by phagocytosis. Finally, therapeutic nanoparticles need to be taken up by the epithelial cells which are connected by tight junctions [5,6,24,25].

Size reduction below about 200 nm as well as hydrophilic surface shielding and zeta potential reduction are commonly considered as beneficial strategies to allow for mucus penetration of nanomaterials [5,6,25,26,27]. In this context, shielding with the hydrophilic, uncharged polymer poly(ethylene glycol) (PEG) has become the extensively investigated gold standard to reduce mucus interactions and facilitate diffusion through the mucus mesh [21,28,29,30,31,32]. Alternatively, the natural, biodegradable anionic polymer HA proved potential to reduce mucoadhesion and was successfully integrated into nanoparticle formulations for local pulmonary delivery [6,33,34,35,36].

Given that the full potential of local pulmonary mRNA therapy remains constrained by these delivery barriers, there is substantial room for the exploration of new carriers. In this study, a diverse set of recently developed lipoamino fatty acid (LAF) xenopeptide (XP) polyplexes [37,38] was evaluated for the first time in the setting of local pulmonary delivery. These double-pH responsive carriers combine two essential domains: (1) one or two small, polar oligoamino acids (OAAs) containing the tetraethylene pentamine motif and (2) two to four apolar, protonatable LAFs bearing central tertiary amines. The former predominantly bind the nucleic acid whereas the latter provide hydrophobic stabilization at neutral pH and induce a drastic polarity switch upon endosomal protonation [37,38]. This triggers spatial re-arrangement and alters phase distribution from the aqueous phase at neutral pH to lipid/water interface at acidic pH [39,40]. These two domains are connected by branching lysines into various topologies, i.e., different covalent arrangements of building blocks, using solid-phase-assisted peptide synthesis yielding sequence-defined, monodisperse macromolecular carriers [37]. Distinct LAF-XPs recently proved high efficiency for mRNA delivery in ultra-low doses even in the presence of full serum, in hard-to-transfect cells in vitro, and also upon intramuscular and intravenous application in vivo [37,38,41]. The most promising LAF-OAA carrier topologies, i.e., U-shapes and bundles, were now selected for evaluation in pulmonary delivery. Additionally, the effect of hydrophilic nanoparticle surface modification was investigated. For this purpose, a recently published PEGylation strategy by introduction of a PEG lipid [42], or an ionic HA coating of lipo-XP polyplexes [43] was applied. This set of mRNA polyplexes was compared in different models and conditions relevant for local pulmonary mRNA delivery, i.e., submerged transfection of Calu-3 and A549 lung epithelial cells in the presence and absence of serum, transfection in mucus producing air–liquid interface culture of Calu-3 cells, and upon intratracheal administration into BALB/c mice. The study clearly revealed that these differing transfection conditions resulted in alternative rankings of best-performing formulations. U-shaped carriers were advantageous in serum-free and ALI conditions, whereas bundles revealed the highest transfection efficiency in submerged cell culture. Hydrophobic modification of the OAA in U-shapes enhanced their efficiency under all transfection conditions. HA or PEG surface modifications did not strongly affect transfections in vitro, whereas surface modification in combination with the hydrophobized U-shape 1843 enhanced their efficiency in vivo, where bundles were again amongst the most efficient carriers. Based on these observations, it is obvious that both the cationizable core of the LAF-XP polyplexes and the surface modification need to be optimized to enhance lung delivery in vivo and to balance the requirements of intracellular fate and uptake.

## 2. Materials and Methods

### 2.1. Materials

CleanCap EGFP mRNA (5moU) encoding an enhanced version of the green fluorescent protein (EGFP) and CleanCap FLuc mRNA (5moU) encoding firefly luciferase (FLuc) was obtained from TriLink BioTechnologies, San Diego, CA, USA. EZ Cap™ Cy5 firefly luciferase mRNA (5-moUTP) was purchased from ApexBio Technology, Houston, TX, USA. A customized F-Luc mRNA12 with ATTO565 labeling of 20% of the moU was ordered from OZ Biosciences, Marseille, France. Stabilized non-immunogenic messenger RNA encoding firefly luciferase (mRNA-FLuc) was provided by Ethris GmbH, Planegg, Germany using their proprietary modification scheme and manufacturing process. The human epithelial adenocarcinoma cell line Calu-3 and the lung carcinoma cell line A549 (CCL-185) were obtained from the American Type Culture Collection, ATCC, Manassas, VA, USA. Dulbecco’s Modified Eagle’s Medium (DMEM) low glucose with sodium bicarbonate, sodium pyruvate and L-glutamine, Minimum Essential Medium Eagle (MEM) with Earle′s salts, L-glutamine and sodium bicarbonate, and fetal bovine serum (FBS) were purchased from Sigma-Aldrich, St. Louis, MO, USA. Penicillin-streptomycin (10,000 U/mL; 10 mg/mL), stable glutamine, and trypsin/EDTA 10× were obtained from PAN-Biotech GmbH, Aidenbach, Germany. PneumaCult™-ALI Medium was bought from Stemcelll Technologies, Vancouver, BC, Canada. Luciferase Cell Culture Lysis 5× reagent and beetle luciferin sodium salt were purchased from Promega, Madison, WI, USA, and ATP from Roche Diagnostics, Mannheim, Germany. GelRed nucleic acid gel stain was obtained from Biotium, Fremont, CA, USA. Agarose, bromophenol blue, coenzyme A trilithium salt, DL-dithiothreitol, glycylglycine, D-Luciferin, sodium chloride (NaCl), potassium chloride (KCl), disodium hydrogen phosphate (Na_2_HPO_2_), potassium dihydrogen phosphate (KH_2_PO_4_) and tris(hydroxymethyl)aminomethane were bought from Sigma-Aldrich, St. Louis, MO, USA. NaOH was purchased from ORG Laborchemie, Bunde, Germany. HEPES was obtained from BIOMOL, Hamburg, Germany. Boric acid, MgCl_2_, and LabTek I slides were purchased from VWR Chemicals, Leuven, Belgium. 3-(4,5-dimethyl-2-thiazolyl)-2,5-diphenyl-2H-tetrazolium bromide (MTT) was purchased from Carl Roth, Karlsruhe, Germany and dimethyl sulfoxide (DMSO) from Fisherscientific, Loughborough, UK. D(+)-Glucose 1-hydrate was purchased from Applichem, Darmstadt, Germany. Glycerol, and ethylenediaminetetraacetic acid (EDTA) disodium salt dihydrate were obtained from Merck, Darmstadt, Germany. Lipofectamine MessengerMAX reagent (Lipo MMAX) was purchased from ThermoScientific, Lancashire, UK. Heparin sodium (5000 I.U./mL) was obtained from B. Braun, Melsungen, Germany. Hyaluronic acid (HA20K) with an average molecular weight of 3.8 × 10^4^ Daltons was obtained from Lifecore Biomedical, Chaska, MN, USA. 1,2-Dimyristoyl-rac-glycero-3-methoxypolyethylene glycol-2000 (PEG-DMG) was purchased from Avanti Polar Lipids, Alabaster, AL, USA. Transwell^®^ 24-well plate inserts (0.33 cm^2^, polyester, 6.5 mm, 0.4 µm) were obtained from Corning, New York, NY, USA. Sterile tissue culture testplates 96F (96-well plates) were obtained from TPP Techno Plastic Products, Trasadingen, Switzerland. Folded capillary cells DTS1070 were bought from Malvern Panalytical Ltd., Worcestershire, UK.

### 2.2. Methods

#### 2.2.1. Synthesis of Carriers

Synthesis and analysis of LAF-XPs was previously described [37,38,39]. In brief, LAFs were synthesized by reductive amination of dodecanal and amino fatty acids, specifically 4-aminobutyric acid for 12Bu and 8-aminooctanoic acid for 12Oc. Sodium cyanoborohydride was used as reducing agent. The reaction was carried out for 48 h in a mixture of dry THF and dry methanol. Purified product was analyzed by ESI-MS [37,39].

The Boc- and Fmoc-protected OAAs were synthesized and purified as previously published with detailed methods for Stp [37] and chGtp as well as Htp [44] in a three-step synthesis. (1) The primary amines of tetraethylene pentamine hydrochloride were protected with trifluoroacetic acid ethyl ester and secondary amines were protected by using Boc anhydride. (2) The primary amines were regenerated by alkaline hydrolysis. (3) The terminal primary amines were asymmetrically modified with a dicarboxylic acid and an Fmoc-protection group. The protected products were purified by dry column vacuum chromatography.

Finally, LAF-XPs were assembled from these purified building blocks and protected L-lysines by using Fmoc-solid-phase synthesis on a 2-chlorotrityl chloride resin as previously described. [37,38] The purified, monodisperse products were analyzed by MALDI-TOF-MS.

#### 2.2.2. Formation of Non-Coated and PEGylated LAF-XP Polyplexes

Nanoparticles were formed as previously described [38] by equal volume mixing of LAF-XP dilution and mRNA dilution. Therefore, LAF-XPs were diluted in purified water at a suitable concentration for indicated N/P (nitrogen/phosphate) ratios under consideration of all primary, secondary, and tertiary amines. In the case of PEGylated nanoparticles, PEG-DMG was first diluted in water and LAF-XP was added subsequently to the same solution. The mRNA was dissolved in HEPES-buffered glucose (HBG) (20 mmol/L of HEPES, 5% (*w*/*v*) glucose, pH 7.4) at a concentration of 25 μg/mL. Mixing was performed by rapid pipetting followed by incubation for 40 min at RT. The final mRNA concentration was 12.5 μg/mL if not stated otherwise. In the case of air–liquid interface cell transfections or in vivo experiments, mRNA was dissolved in HBG with double amount of glucose (20 mmol/L of HEPES, 10% (*w*/*v*) glucose, pH 7.4) to obtain a final isosmotic formulation and mRNA concentration was adjusted to reach the indicated mRNA concentration after equal volume mixing. Control mRNA formulations with commercial lipofectamine were prepared according to manufacturer’s instructions in Opti-MEM at an mRNA concentration of 10 µg/mL with 0.15 µL Lipofectamine MessengerMAX (Lipo MMAX) reagent per 0.1 µg mRNA.

#### 2.2.3. Hyaluronic Acid (HA) Coating of LAF Xenopeptide Polyplexes

Non-coated core LAF-XP polyplexes were formed as described above in 80% of the final desired polyplex solution volume at a mRNA concentration of 15.6 µg/mL. Hyaluronic acid (HA20K) was diluted in HBG buffer to a volume of 20% of the final desired polyplex solution volume. Indicated equivalents of hyaluronic acid monomers per equivalent of carrier were used. After 40 min of incubation at RT, the uncoated LAF-XP polyplexes were quickly added to the hyaluronic acid solution and mixed by careful pipetting. The mixture was incubated for 10 min at room temperature to form coated nanoparticles at a final mRNA concentration of 12.5 µg/mL. In case of HA titrations, uncoated LAF-XP polyplexes were prepared similarly but diluted in HBG without HA in the last step.

#### 2.2.4. Size and Zeta Potential

LAF-XP polyplexes (40 µL) were prepared with mRNA-FLuc (CleanCap FLuc mRNA (5moU), TriLink BioTechnologies, San Diego, CA, USA) as described for HA coated particles, if not indicated differently. Size and zeta potential were measured with a Zetasizer Nano ZS (Malvern Panalytical Ltd., Worcestershire, UK) in a folded capillary cell (DTS1070) by dynamic and electrophoretic light scattering (DLS, ELS). For size and polydispersity index (PdI) measurements, the samples were diluted with HBG ad 80 µL and three measurements with six sub runs were performed. Subsequently, these samples were diluted further ad 800 μL with HBG directly before zeta potential measurement with three automatic measurements with 5 to 15 sub runs each. The following instrument settings were used: equilibration time 30 s (size, PdI) or 60 s (zeta potential), temperature 25 °C, refractive index 1.330, viscosity 0.8872 mPa×s, dielectric constant 78.5. Zeta potentials were calculated using the Smoluchowski equation.

#### 2.2.5. Agarose Gel Shift Assay

An agarose gel shift assay was performed as reported previously [38]. In brief, a gel with 1% (*w*/*v*) agarose in TBE buffer (18.0 g of tris(hydroxymethyl)aminomethane, 5.5 g of boric acid, 4 mL of 0.5 mol/L ethylenediamine tetraacetic acid (EDTA) at pH 8, in 1 L of water) was prepared and stained with GelRed. LAF-XP polyplexes were formed as described for HA-coated particles with mRNA-FLuc (CleanCap FLuc mRNA (5moU), TriLink BioTechnologies, San Diego, CA, USA). A 6× loading buffer (6 mL of glycerol, 1.2 mL of 0.5 mol/L EDTA at pH 8.0, 2.8 mL of water, 0.02 g of bromophenol blue) was diluted 1:6 to 20 μL of LAF-XP polyplex solution. Thereof, gel electrophoresis in TBE buffer was performed (120 V, 70 min). Free mRNA-FLuc in a 1:1 mixture of purified water and HBG (12.5 μg/mL) served as control.

#### 2.2.6. ATTO643 Labeling

Labeling of DBCO-modified HA with azido-functionalized ATTO643 dye (HA-ATTO643) was described in Vetter et al. 2025 [43].

#### 2.2.7. Fluorescence Cross Correlation

Core LAF-XP polyplexes were formed as described for HA-coated particles at an mRNA concentration of 83.3 µg/mL (2.5% (*w*/*w*) mRNA-ATTO565, 97.5% (*w*/*w*) mRNA-FLuc without label). Particles were coated with a mix of unlabeled HA and HA-ATTO643 at an ATTO643/ATTO565 ratio of 2, yielding a final mRNA concentration of 66.7 µg/mL. The labeled polyplexes were measured in a 1:10 dilution in HBG after preparation. As a control, freely diffusing mRNA-ATTO565 and HA-ATTO643 were measured. The experiments were performed in 8-well LabTek I slides (Nunc™ Thermo Fisher Scientific, Darmstadt, Germany), using approximately 20 µL of each sample. The data were acquired on a home-built laser-scanning confocal microscope (AG Lamb, LMU Munich, Germany), described elsewhere [45,46] and routinely checked for alignment using a 10 nM mixture of ATTO565-COOH and ATTO655-COOH (ATTO-TEC now Leica Microsystems, Wetzlar, Germany) in dPBS (Gibco^TM^ Thermo Fisher Scientific, Darmstadt, Germany). The experiments were performed at room temperature with a 60× water immersion objective (Plan Apo 60× WI/NA 1.27, Nikon, Tokyo, Japan), focusing 15–20 µm above the surface. The excitation was achieved using a 560 nm-pulsed diode laser (LDH-P-FA-560, PicoQuant, Berlin, Germany 2 µW before the objective) for ATTO565-labeled species and a 635 nm-pulsed diode laser (LDH-P-C-635M, PicoQuant, Berlin, Germany, 1 µW before the objective) for ATTO643-labeled species. The lasers were pulsed at a frequency of 25 MHz and the red laser was electronically delayed by ~17 ns, to achieve pulsed interleaved excitation (PIE) and minimize the cross-talk [47]. The fluorescence emission was recorded for 10 min by two avalanche photodiode detectors (SPCM-AQR-14, Perkin Elmer, Shelton, CT, USA, after a 595/50 bandpass emission filter for ATTO565 and Count^®^ Single Photon Counting Module, Laser Components, Olching, Germany, after 635 nm longpass emission filter for ATTO643). Time-correlated single-photon-counting electronics (TCSPC cards, SPC-150 Becker and Hickl, Berlin, Germany) were used to precisely synchronize excitation and emission. The raw photon data were analyzed with the software PIE Analysis in MATLAB (PAM, v1.6 available at https://gitlab.com/PAM-PIE (accessed on 20 May 2026) and operated with MATLAB 2023b) to compute auto- and cross-correlations [48]. Depending on the sample, the autocorrelation functions (ACFs) were fit with a model containing one or two diffusional components. Assuming a 3D Gaussian focus shape, the ACF is given by:

(1) $$G\left(\tau \right)=\frac{\gamma }{{\left(A_{1}+A_{2}\right)}^{2}}\left\{\left[A_{1}{\left(1+\frac{4D_{1}\tau }{\omega_{r}^{2}}\right)}^{-1}{\left(1+\frac{4D_{1}\tau }{\omega_{z}^{2}}\right)}^{-\frac{1}{2}}\right]+\;\left[A_{2}{\left(1+\frac{4D_{2}\tau }{\omega_{r}^{2}}\right)}^{-1}{\left(1+\frac{4D_{2}\tau }{\omega_{z}^{2}}\right)}^{-\frac{1}{2}}\right]\right\}$$

Here, the time lag of the correlation is denoted as τ, $D_{i}$ the diffusion coefficient of the species i, and $\omega_{r}$ and $\omega_{z}$ are respectively the lateral and axial dimension of the observation volume. γ is a geometrical correction factor used to account for the shape of the 3D Gaussian focus and is equal to 2^−3/2^. $A_{i}$ is the amplitude of species i within the observation volume. This model assumes equal molecular brightness for all species. When this assumption is respected, $A_{i}$ is equal to the particle number $N_{i}$ and the average molecular brightness is defined as the fluorescence intensity divided by the sum of $N_{i}$. When this condition is not met, $A_{i}$ can only be used as an estimation of $N_{i}$. Accurate quantification of $N_{i}$ requires including the relative brightness of each species in the analyses [49]. When measuring polyplexes, determining the brightness of each species is particularly challenging, as individual particles might incorporate multiple fluorescent labels in a stochastic manner. Therefore, an accurate quantification of the molecular brightness and/or of $N_{i}$ could not be achieved.

Similarly, the fluorescence cross-correlation function (CCF) is given by:

(2) $$G\left(\tau \right)=\frac{\gamma \;A_{YR}}{\left(A_{YT}\right)\left(A_{RT}\right)}\left[{\left(1+\frac{4D_{YR}\tau }{\omega_{r}^{2}}\right)}^{-1}{\left(1+\frac{4D_{YR}\tau }{\omega_{z}^{2}}\right)}^{-\frac{1}{2}}\right]$$

where $A_{YR}$ is the amplitude of double-labeled particles and $A_{YT}\;$ and $A_{RT}\;$ represent the total number of yellow-labeled and red-labeled particles, respectively. Also, in the case of the CCF, $A_{i}$ can only be used as an estimation of $N_{i}$ if the brightnesses of the individual species are not equal.

#### 2.2.8. Cell Culture

The human lung cancer cell line A549 was cultured in Dulbecco’s Modified Eagle’s Medium (DMEM)-low glucose-(1 g/L glucose)-containing L-glutamine, sodium bicarbonate and sodium pyruvate supplemented with 10% FBS, 100 U/mL of penicillin, and 100 μg/mL of streptomycin. The human lung adenocarcinoma cell line Calu-3 was cultured in antibiotics-free Minimum Essential Medium Eagle (MEM) with Earle′s salts, L-glutamine and sodium bicarbonate supplemented with 10% FBS at submerged conditions. The cells were cultured and incubated at 37 °C and 5% CO_2_ at a relative humidity of 95% if not stated otherwise.

#### 2.2.9. Luciferase Expression Assay

For submerged conditions, cells were seeded in 96-well plates at numbers of 30,000 cell/well for Calu-3, or 20,000 cells/well for A549 cells one day before the transfection. Prior to transfection, cell culture medium was replaced with 99 µL fresh culture medium per well. In the case of serum-free transfections, culture medium was replaced with serum-free medium. LAF-XP polyplexes were formed with mRNA-FLuc (CleanCap FLuc mRNA (5moU), TriLink BioTechnologies, San Diego, CA, USA) as described above (12.5 µg/mL mRNA-FLuc) at indicated N/P ratios. LAF-XP polyplex solution (1 µL) was added to each well to evaluate transfection efficiency at a dose of 12.5 ng mRNA per well. HBG buffer was used as negative control. The transfection medium was removed after 4 h and replaced by culture medium supplemented with 10% FBS, only in the case of serum-free transfection studies in both experimental conditions, i.e., serum-free and control serum samples. After incubation (24 h, 37 °C), the medium was removed, cells were lysed with 100 μL of cell culture 0.5× lysis buffer, and frozen at −80 °C at least overnight. Before measurement, plates were incubated on a rocking shaker (1 h, RT, 25 rpm). Cell lysates were 1:100 diluted in phosphate-buffered saline (PBS) (8 g NaCl, 0.2 g KCl, 1.15 G Na_2_HPO_4_, and 0.2 g KH_2_PO_4_ in 1 L purified water) and luciferase activity was measured for 10 s in 35 µL lysate dilution using a Centro LB 960 microplate luminometer (Berthold Technologies, Bad Wildbad, Germany) after the addition of 100 μL LAR buffer (20 mmol/L glycylglycine, 1 mmol/L MgCl_2_, 0.1 mmol/L EDTA, 3.3 mmol/L dithiothreitol, 0.55 mmol/L adenosine 5′-triphosphate, 0.27 mmol/L coenzyme A, pH 8.0–8.5) supplemented with 5% (*v*/*v*) of a mixture of 10 mmol/L luciferin-sodium and 29 mmol/L glycylglycine. Transfection efficiency was calculated as relative light units (RLU) per number of seeded cells per well after background subtraction (i.e., RLU values of HBG-treated cells). Experiments were performed in triplicates.

#### 2.2.10. Air–Liquid Interface

For air–liquid interface (ALI) conditions, Calu-3 cells were seeded at ALI cell culture following Rademacker et al. prior to transfection [50]. Briefly, cells were detached using 0.25% trypsin-EDTA and seeded at a density of 250,000 cells per insert and incubated. After 3 days, apical medium was aspirated to obtain an air–liquid interface. Cells were cultured for at least one week, with medium replenishment every 2–3 days with PneumaCult™-ALI Medium. LAF-XP polyplexes were formed with mRNA-FLuc (CleanCap FLuc mRNA (5moU), TriLink BioTechnologies, San Diego, CA, USA) at indicated N/P ratios at an mRNA concentration of 25 µg/mL. For transfection, 24 µL LAF-XP polyplex solution was carefully applied onto the mucus surface to evaluate transfection efficiency at a dose of 300 ng mRNA per well. After 24 h incubation at 37 °C, the transfection medium was removed, cells were washed twice with PBS and directly lysed with 100 μL of cell culture 1× lysis buffer per well (1 h, RT) on a rocking shaker (30 rpm). Cell lysates were 1:10-diluted in PBS and luciferase expression was measured as described above.

#### 2.2.11. Flow Cytometry

To evaluate EGFP expression in Calu-3 cells under air–liquid interface conditions, LAF-XP polyplexes were formulated as described above with mRNA-EGFP (CleanCap FLuc mRNA (5moU), TriLink BioTechnologies, San Diego, CA, USA) at a final mRNA concentration of 25 µg/mL. Transfection was performed analogously to the luciferase expression assay at a dose of 600 ng mRNA per well (i.e., 24 µL). Cells were incubated for 24 h (37 °C). After transfection, the solution was removed and cells were detached from the insert by scraping in 100 µL PBS. The cell suspension was centrifuged, the supernatant was discarded, and the pellet was washed once with PBS followed by a second centrifugation step. The final pellet was resuspended in PBS containing 2 mM EDTA, briefly vortexed, and analyzed by flow cytometry using an Attune NxT instrument (Thermo Fisher Scientific, Darmstadt, Germany).

To evaluate cell association and uptake in Calu-3 cells under air–liquid interface conditions, LAF-XP polyplexes were formulated with 80% (*w*/*w*) mRNA-EGFP (CleanCap FLuc mRNA (5moU), TriLink BioTechnologies, San Diego, CA, USA) and 20% (*w*/*w*) Cy5-mRNA (EZ Cap™ Cy5 firefly luciferase mRNA (5-moUTP), ApexBio Technology, Houston, TX, USA), yielding a final mRNA concentration of 25 µg/mL. LAF-XP polyplexes at a dose of 600 ng mRNA per well (i.e., 24 µL) were added onto the cells, followed by 6 h incubation (37 °C). Afterwards, transfection solution was removed, cells were harvested as described earlier and analyzed using an Attune NxT instrument (Thermo Fisher Scientific, Darmstadt, Germany).

For submerged conditions, 30,000 Calu-3 cells seeded one day prior to transfection were transfected without medium change by addition of 2 µL LAF-XP polyplexes (i.e., 25 ng mRNA) formed with 80% (*w*/*w*) mRNA-EGFP (CleanCap FLuc mRNA (5moU), TriLink BioTechnologies, San Diego, CA, USA) and 20% (*w*/*w*) Cy5-mRNA (EZ Cap™ Cy5 firefly luciferase mRNA (5-moUTP), ApexBio Technology, Houston, TX, USA) at a total mRNA concentration of 12.5 µg/mL. After 4 h incubation (37 °C), the material was processed as reported in [38]. In short, after medium removal cells were incubated on ice with cold heparin solution (1000 I.U./mL in PBS) for 15 min to remove unspecifically surface-bound nanoparticles, followed by two washing steps with PBS and detachment with 1× trypsin/EDTA. Cells were then resuspended in a solution of 10% FBS in PBS supplemented with DAPI (1 µg/mL) and transferred to a flat-bottom plate (Thermo Fisher Scientific, Roskilde, Denmark). Measurements were performed with a CytoFLEX S flow cytometer (Beckman Coulter, Brea, CA, USA) with excitation/emission of Cy5 at λ = 638/660 nm and EGFP at λ = 488/532 nm. Cells were gated based on their forward- and side-scatter profile and at least 880 live cells per well were evaluated. Experiments were performed in triplicates. Data was analyzed with FlowJo 10.6.0 software (FlowJo, Ashland, OR, USA) and presented as percentage of transfected cells of the single, live cell population for each wavelength. MFI(Cy5) was evaluated for the Cy5+ population, while MFI(EGFP) values refer to the live cell population.

#### 2.2.12. In Vivo Intratracheal Administration

All procedures were approved by the local animal welfare authorities (Regierung von Oberbayern; ROB-55.2-2532.Vet_03-23-28) and were conducted according to the German animal protection law (Tierschutzgesetz).

#### 2.2.13. Animal Housing

BALB/c mice (Janvier, Le Genest-Saint-Isle, France) were housed under specific pathogen-free conditions (facility tested negative for any FELASA-listed pathogens according to the annual health and hygiene survey) in individually ventilated cages under a circadian light cycle (lights on from 7 a.m. to 7 p.m.). Food and drinking water were provided ad libitum. After arrival, animals were given at least 7 days for acclimatization until they entered the study.

#### 2.2.14. Particle Formation for In Vivo Experiments

Particles were formed as described in Section 2.2.2 and Section 2.2.3 with the following changes: mRNA-FLuc kindly provided by Ethris GmbH, Planegg, Germany was used. The mRNA was dissolved in HBG with 10% (*w*/*v*) glucose to obtain a final isosmotic formulation. The final mRNA concentration of all in vivo formulations was 0.06 mg/mL. The LAF-XP polyplexes were freshly prepared for in vivo experiments and checked by DLS/ELS with 20–30 µL of sample as described above, as an in process control.

#### 2.2.15. Intratracheal Instillation

An amount of 50 µL (c(mRNA) = 0.06 mg/mL) of the formulations was applied by intratracheal instillation (i.e., pipetted in one bolus on the tip of an intratracheal 20 G tubus and aspirated by the animal during a physiological inspiratory movement) under short- time Isoflurane inhalation anesthesia to nine-week-old BALB/c mice.

#### 2.2.16. Necropsy and Ex Vivo Bioluminescence Imaging

Mice (*n* = 5; 1841 *n* = 4) were anesthetized approximately 6 h after application of the LAF-XP polyplexes by intraperitoneal injection of medetomidine/midazolam/fentanyl (0.5/5.0/0.05 mg/kg bw). Next, 1.5 mg D-luciferin (dissolved in 50 µL PBS) was applied intranasally and 3 mg D-luciferin (dissolved in 100 µL PBS) was injected intraperitoneally. In vivo luciferase activity was captured after an incubation time of 10 min using an IVIS100 In Vivo Imaging System (highest stage, middle binning, record time of 1 min). Subsequently, animals were killed by cervical dislocation. The abdominal and thoracal cavity was opened. The left kidney artery was dissected, and the small circulation was flushed using PBS through the right ventricle of the heart. Lungs, liver, heart, right kidney, and spleen were explanted and placed on a Petri dish. Subsequently, ex vivo luciferase activity was measured using the same settings as for in vivo luciferase activity measurement. Afterwards, the lung (including trachea and bifurcation), liver, heart, right kidney, and spleen were snap-frozen and stored at −80 °C until further processing.

#### 2.2.17. Luciferase Detection in Lung Homogenates

The entire explanted lungs (including bifurcation and trachea) were weighed and then homogenized in lysis buffer using a FastPrep^®^-24 Homogenisator (MP Biomedicals, Eschwege, Germany). A total of 100 μL D-luciferin buffer (3 mg/100 μL PBS) was added automatically by the Lumat LB 9507 Luminometer (Berthold Technologies, Bad Wildbad, Germany) to 75 μL of centrifuged lysates. Luciferase activity was measured as RLU/s and evaluated and depicted as log(RLU/organ).

#### 2.2.18. Statistical Analysis

Results are presented as arithmetic mean with indication of standard deviation if not otherwise stated. Indicated statistical tests were performed with GraphPad Prism 10.6.1 (GraphPad Software, Boston, MA, USA).

## 3. Results and Discussion

### 3.1. Carrier Selection and Polyplex Formation

In double-pH-responsive LAF-XP carriers, OAAs containing the tetraethylene pentamine motif are connected by lysines via solid-phase peptide synthesis in a sequence-defined manner with apolar protonatable LAFs of different chain lengths and positions of tertiary amines. This strategy yielded a library with a multitude of possible combinations and topologies whereby each LAF-XP exhibits an exact, monodisperse structure and molecular weight [37,38,39]. High batch-to-batch reproducibility of the synthesis and biological activity had been previously demonstrated [38]. For distinct nucleic acid cargos, beneficial topologies with respective optimal types and ratios of LAFs and OAAs were identified [37]. In the case of mRNA delivery, U-shape carriers with an LAF/OAA ratio of 2:1 (U-2:1) and medium LAF chain lengths and terminal alkyl chains as well as bundles (B-4:1) with an LAF/OAA ratio of 4:1 and shorter LAFs were most beneficial for particle formation and transfection efficiency [37,39]. In this study, the most promising carriers for mRNA delivery were selected for evaluation in topical pulmonary delivery.

**Scheme 1:**
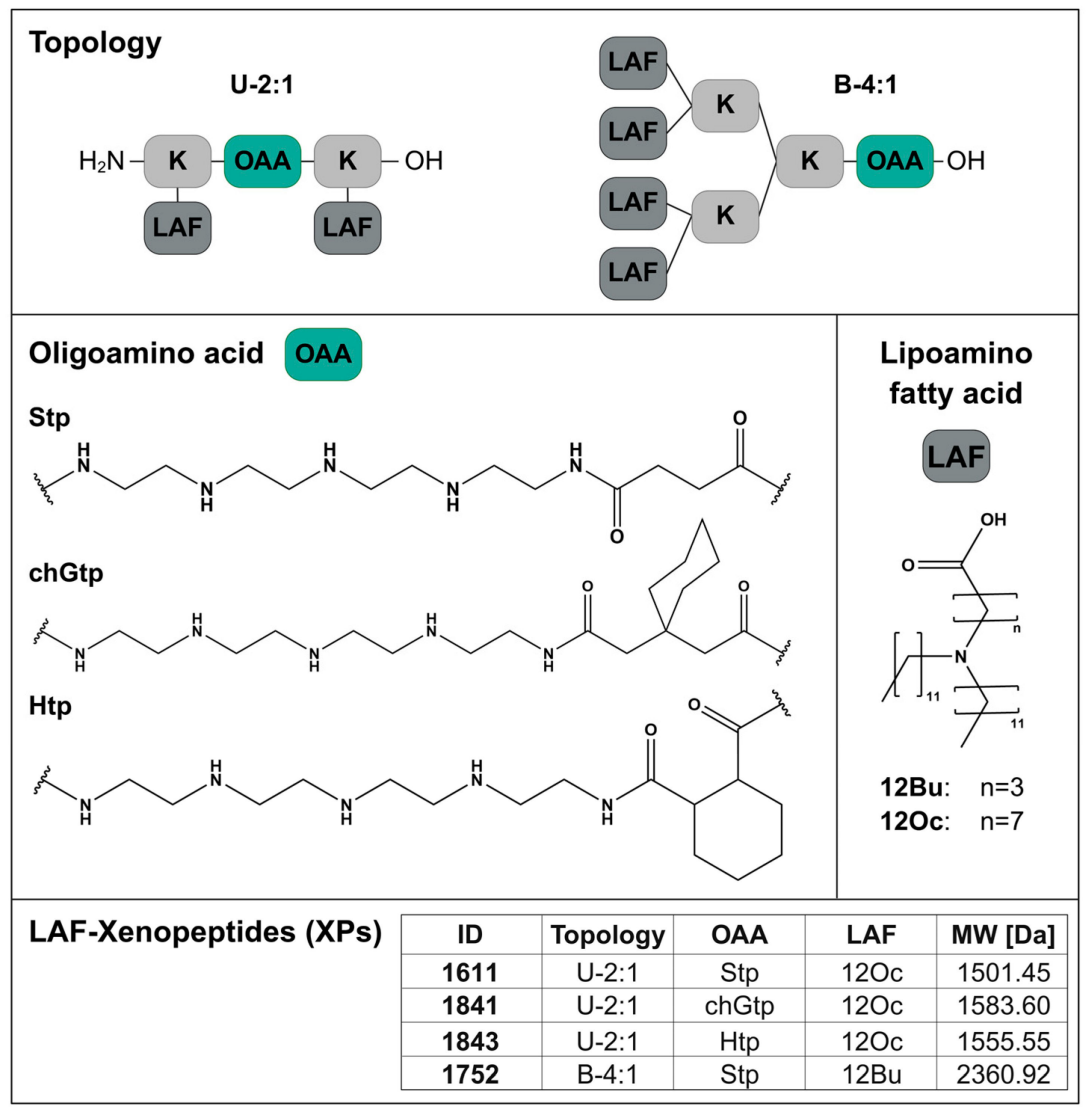
Schematic structures, building blocks, identification (ID) numbers, and molecular weight (MW) of sequence-defined lipoamino fatty acid (LAF)-xenopeptides (XPs) evaluated in the following experiments. K, lysine; OAA, oligoamino acid; Stp, succinoyl tetraethylene pentamine; chGtp, 3-(cyclohexyl)glutaroyl tetraethylene pentamine; Htp, hexahydrophthaloyl tetraethylene pentamine; LAF, lipoamino fatty acid; 12Oc, 8-(didodecylamino)octanoic acid; 12Bu, 4-(didodecylamino)butyric acid. The nomenclature indicates the topology (U: U-shape, B: bundle) and the LAF/OAA ratio (2:1, 4:1). The LAF-XPs 1611 and 1752 were synthesized and analyzed as described in Thalmayr et al. and Grau et al. [37,39] and 1841 and 1843 as described in Burghardt et al. [38].

This set, on the one hand, comprises said U-shape carriers (U-2:1). U-2:1 with ID number 1611, bearing two times the LAF 8-(didodecylamino)octanoic acid (12Oc) with one succinoyl tetraethylene pentamine (Stp) (Scheme 1) showed high mRNA encapsulation efficiency and formation of small, stable particles [37,39,41]. Carrier 1611 previously demonstrated mRNA transfection efficiency after i.v. and i.m. administration and was tolerated in repeated injections (*n* = 3) [37,41]. Hydrophobization of the OAA domain in these U-shape carriers by the introduction of hydrocarbon moieties increased endosomal release and enhanced in vitro and in vivo mRNA expression in previous work. The OAAs hexahydrophthaloyl tetraethylene pentamine (Htp) and 3-(cyclohexyl)glutaroyl tetraethylene pentamine (chGtp) (Scheme 1) were especially promising units of U-shape carriers 1843 and 1841, respectively [38].

On the other hand, bundle topology B-4:1 with four times the LAF 4-(didodecylamino)butyric acid (12Bu) (Scheme 1) and Stp (1752) was chosen due to its potent endosomal escape, high efficiency in ultra-low doses in the presence of serum and a high in vivo efficiency [37,41]. These carriers are more hydrophobic due to their LAF/OAA ratio of 4:1 and form slightly bigger, colloidally less-stable particles. Stp was the most efficient OAA for this topology [38].

In sum, U-2:1 topology with Stp (1611), Htp (1843) and chGtp (1841) as OAA domain and 12Oc as LAF, as well as B-4:1 with Stp and 12Bu (1752) were selected (Scheme 1). The mRNA nanoparticles were formed by turbulent flash mixing via rapid pipetting of equal volumes of mRNA diluted in HEPES-buffered glucose (HBG, 5% (*w*/*v*) glucose, pH 7.4) and LAF-XPs diluted in purified water. The ratio of carrier to mRNA was defined by the ratio of all protonatable primary, secondary, and amines of the carriers to phosphates of the mRNA backbone (N/P ratio). Previously published optimum N/P ratios for particle formation, stability and transfection efficiency were applied: N/P 18 for U-2:1 and N/P 24 for B-4:1 [37,38]. Noteworthy, these N/P ratios do not reflect charge ratios as only a fraction of secondary amines per OAA is protonated at neutral pH [51]. It cannot be excluded that an excess of carrier to mRNA might result in free or micellar LAF-XPs that favorably support cellular uptake and transfection efficiency, as described for PEI [52,53].

### 3.2. PEGylation

**Scheme 2:**
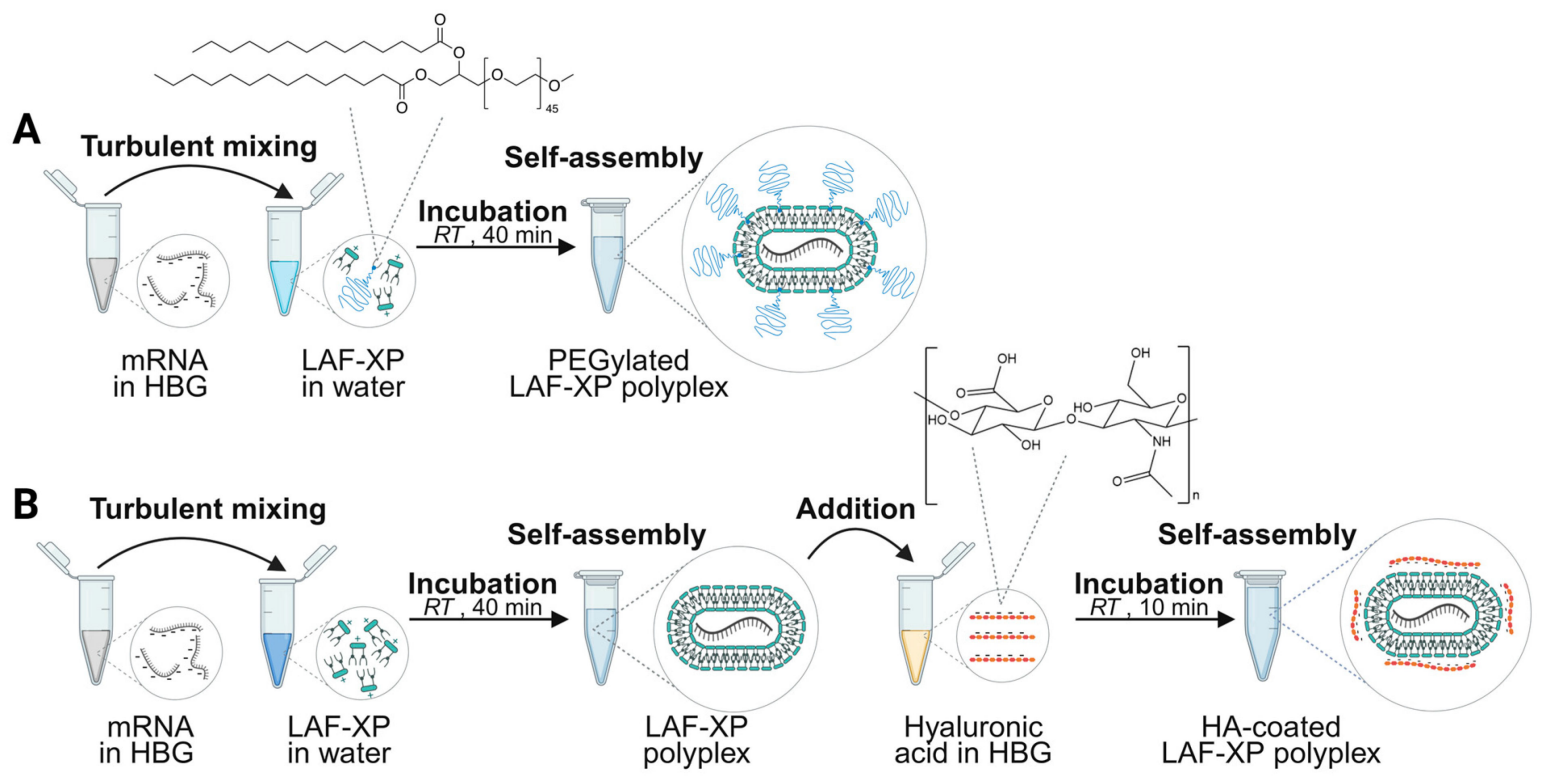
Particle formation with surface modification. (**A**) PEG-DMG was diluted in water followed by addition of LAF-XP. The resulting solution was mixed by rapid pipetting with mRNA diluted in HBG at distinct N/P ratios followed by 40 min incubation at RT. (**B**) Equal volumes of LAF-XPs diluted in water and mRNA diluted in HBG were mixed at distinct N/P ratios by rapid pipetting. After 40 min incubation at RT, LAF-XP polyplexes were added to indicated amounts of hyaluronic acid (HA) in HBG in a volume-mixing ratio of 8/2 (polyplex-solution/HA-solution), carefully mixed and incubated at RT for another 10 min. Depiction of one HA monomer, that is, β-glycosidically linked D-glucuronic acid and N-acetyl-D-glucosamine. *n* = 55–105 for HA20K (Lifecore Biomedical, Chaska, MN, USA).

PEGylation of nanoparticles is a widely applied strategy to enhance mucus penetration and to improve local pulmonary drug delivery [6,21,28,29,30,32]. Incorporation of low amounts of 3% PEG-DMG into LAF-XP polyplexes via lipid anchoring (Scheme 2A) provides increased colloidal particle stability under serum and salt stress, while maintaining high mRNA transfection levels, and increasing tolerability at higher doses in vivo. With increasing amounts of PEG, particle size decreased. Molar ratios of PEG-DMG over 10% reduced the zeta potential by over one third at the expense of reduced efficiency [42]. This phenomenon is known as “PEG dilemma”, as PEGylation not only can stabilize particles but also shields against membrane interactions required for cellular uptake and endosomal escape [54,55,56]. Herein, U-12Oc:Stp-2:1 (1611) and B-12Bu:Stp-4:1 (1752) were modified with low and moderate PEGylation ratios of 3% and 10%, yielding colloidally stabilized but not completely shielded particles <90 nm or <75 nm, respectively, with moderately positive zeta potential <17 mV [42]. These mRNA nanoparticles with hydrophilic surface modification were subsequently employed to experiments in lung delivery models.

### 3.3. Hyaluronic Acid Coating

**Figure 1:**
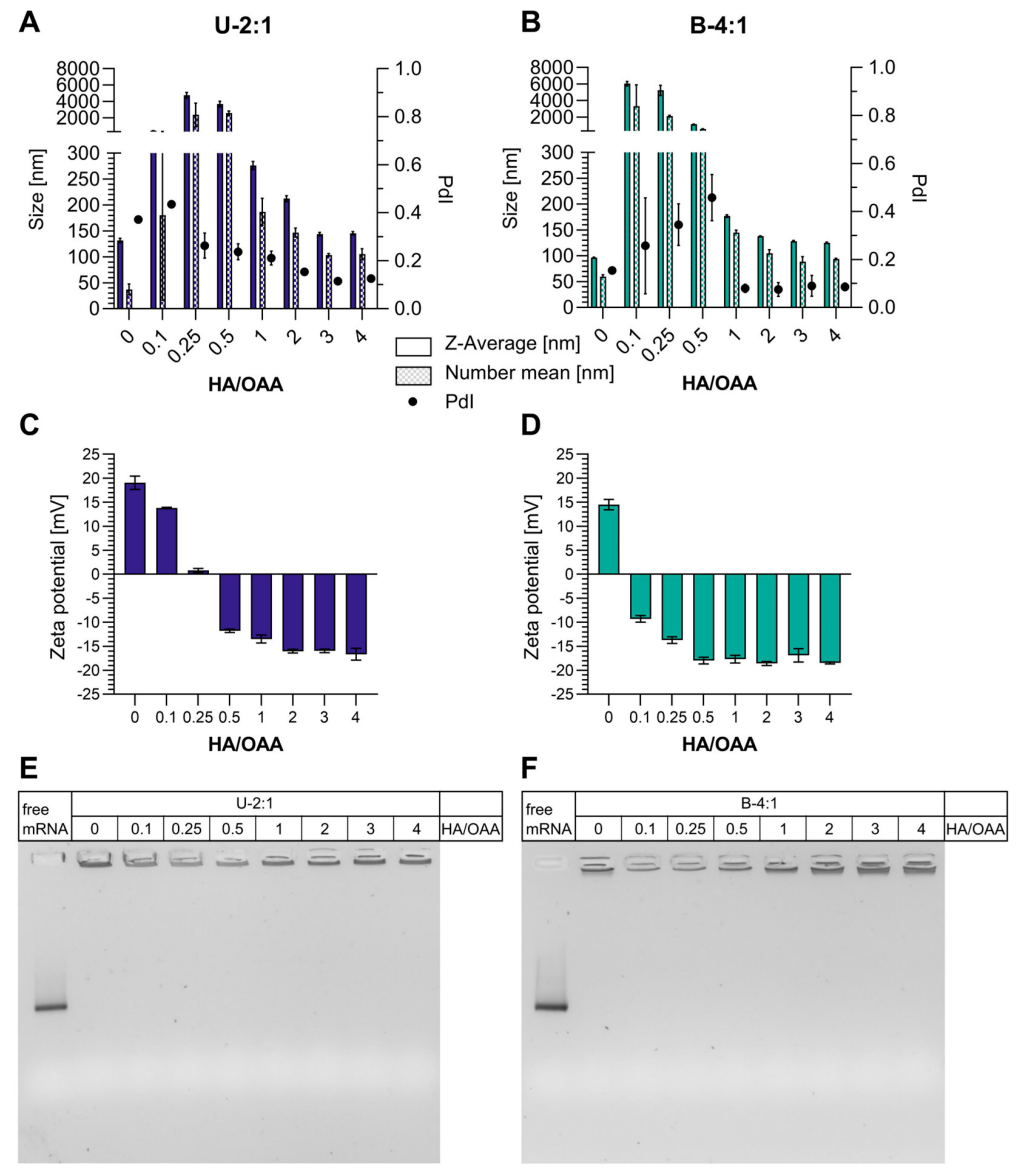
Hyaluronic acid (HA) titration with LAF-XP polyplexes. (**A**,**B**) Z-Average, number mean, PdI and (**C**,**D**) zeta potential of LAF-XP polyplexes modified with indicated molar ratios of hyaluronic acid monomers per Stp-unit (HA/OAA). (**E**,**F**) Respective gel shift assays with free mRNA-FLuc (length: 1922 nucleotides) as control for non-encapsulated cargo. (**A**,**C**,**E**) U-2:1 (1611, OAA = Stp) at N/P 18; (**B**,**D**,**F**) B-4:1 (1752, OAA = Stp) at N/P 24.

HA is a natural, biodegradable, and negatively charged glucosamino glycane abundant in the extracellular matrix [57]. It has been exploited as shielding agent-masking positively charged nanoparticles [58,59,60]. Also, it is known to bind to CD44 receptors which makes it an interesting targeting ligand for CD44-overexpressing cells [57,61,62,63,64,65,66,67]. Besides PEGylation, HA coating was found to enhance mucus penetration in several studies [6,33,34,35,36,62]. Strategies for HA functionalization of nanoparticles include covalent attachment [61], lipid anchoring [36,62,63,64,65,68], and surface coating by electrostatic interactions between the anionic HA and the positively charged particle surface [11,43,66,67,69,70,71,72]. Herein, an ionic coating strategy was applied to Stp-based LAF-XP polyplexes of two different topologies, i.e., 1611 (U-2:1) and 1752 (B-4:1) (Scheme 2B). The particle core was formed by equal volume flash mixing of LAF-XPs diluted in water with mRNA diluted in HEPES-buffered glucose (HBG) in 80% (*v*/*v*) of the final volume. After 40 min incubation at *RT*, LAF-XP polyplexes were quickly added to HA diluted in HBG and incubated for another 10 min. A titration of hyaluronic acid, from 0 to 4 equivalents of HA monomers per equivalent of OAA, was performed (Figure 1). This ratio represents a charge ratio of negatively charged carboxyl groups to positively charged secondary amines of the OAA at neutral pH and is referred to as HA/OAA ratio in the following. DLS data showed particle aggregation for HA/OAA ratios below <1 and formation of defined particles starting from a ratio of 1 for both topologies (Figure 1A,B). The size increased compared to non-coated particles. Low polydispersity (PdI < 0.2) was achieved starting from an HA/OAA ratio of 1 or 2 for the bundle and U-shape, respectively.

A surface charge inversion from positive to negative zeta potential was already observed at an HA/Stp ratio of 0.5 in the case of U-shape 1611 and a ratio of 0.1 in the case of bundle 1752 (Figure 1C,D). This seems reasonable, as only a fraction of secondary amines per OAA is protonated at neutral pH [51] and positive charges are already partially compensated by negatively charged carboxylates of the carrier and phosphates of the mRNA. Additionally, the protonated amines are only partially exposed to the particle surface. U-shape carriers required higher HA/Stp ratios compared to bundles to reach charge conversion (Figure 1C,D). This might be attributed to the additional protonated primary amine of U-shapes, yielding a higher charge per carrier for U-shapes than for bundles at neutral pH. Size and zeta potential reached a plateau starting from an HA/Stp ratio of 3 (Figure 1A–D) with PDI ~0.1 and zeta potentials around −17 mV. Gel shift assays (Figure 1E,F) were performed to analyze whether the mRNA cargo was fully complexed or released due to the presence of competing anionic HA polymers. While mRNA/LAF-XP complexes would be retained in the pockets, free mRNA would move through the gel. The absence of bands of free mRNA indicated full cargo complexation at all HA/Stp ratios for both carriers. In sum, HA/OAA of three was used in further experiments yielding full mRNA binding in low-dispersity nanoparticles of ~130–150 nm with HA saturation. This is in line with the previously published data by Vetter et al. where HA was coated onto siRNA polyplexes made of T-shaped lipo-XPs with oleic acid side chains [43]. Despite a notable size increase for HA-coated particles compared to unmodified LAF-XP polyplexes (Figure 1A,B), the coated particles remain below the reported size-cutoff of 200 nm [6,27] or up to 500 nm [30] required for effective mucus penetration.

Fluorescence correlation and cross-correlation spectroscopy (FCS and FCCS) investigate the mobility and interactions of two differently fluorescently labeled molecules by examining the intensity fluctuations caused by their (co-)diffusion through a confocal observation volume [73,74]. The intensity fluctuations are recorded simultaneously in two detection channels, each corresponding to one label, and are first analyzed separately with an autocorrelation function (ACF). The temporal decay of the ACF reflects the diffusion of the molecule, with a slower diffusion shifting the ACF decay towards longer time scales. The intensity fluctuations of the two detection channels are then jointly analyzed with a cross-correlation function (CCF), which yields a positive signal only when the two labeled species are interacting and co-diffusing. The amplitude of the CCF is directly proportional to the number of dual-labeled, interacting species.

**Figure 2:**
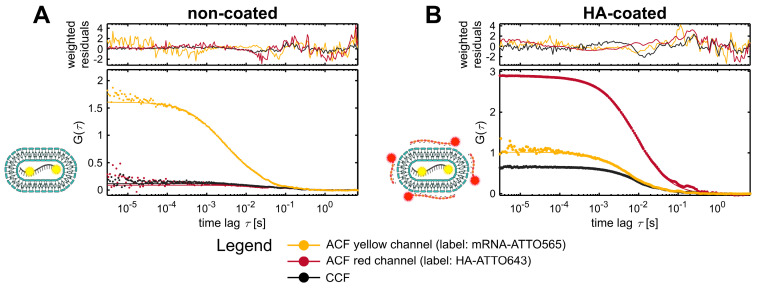
Fluorescence cross correlation spectroscopy (FCCS). FCCS of (**A**) non-coated or (**B**) HA-coated 1611 LAF-XP polyplexes (3 HA/OAA). Measurement in HBG buffer directly after particle formation. The fit parameters are reported in supporting Table S1. Red: HA-ATTO643 auto-correlation function (ACF); yellow: mRNA-ATTO565 ACF; black: cross-correlation function (CCF).

The method was used to confirm the HA-functionalization of LAF-XP polyplexes (HA/OAA = 3) exemplary for U-shape 1611 [43,75]. Fluorescently labeled polyplexes were prepared by mixing-unlabeled mRNA with 2.5% *w*/*w* yellow-labeled mRNA-ATTO565, and HA-coated polyplexes were formulated with red labeled HA-ATTO643 at an ATTO643/ATTO565 ratio of 2. The polyplexes were measured in HBG right after a 1:10 dilution in HBG. The ACFs of the yellow channel (560 nm excitation, ATTO565-labeled species) and red channels (635 nm excitation, ATTO643-labeled species) were analyzed using a one or two-component diffusion model to account for potential release of mRNA and HA, whose respective diffusion coefficients were established by measuring free mRNA-ATTO565 and free HA-ATTO634 as controls (Table S1). Directly after particle formation, unmodified particles showed a yellow ACF decaying in the millisecond regime, confirming proper mRNA encapsulation and a neglectable red ACF and no cross correlation, as expected due to the absence of a second label (Figure 2A). For HA-coated particles, the analysis revealed cross correlation between the two labels as indicated by the high cross correlation function (CFF) amplitude which demonstrated the retention of HA on the particle (Figure 2B). The incorporation of HA is further confirmed by the similar decay profile of both ACFs and CCF. These data corroborate the effective coating of HA onto LAF-XP mRNA polyplexes.

### 3.4. Transfection of Lung Epithelial Cells Under Submerged Conditions

**Figure 3:**
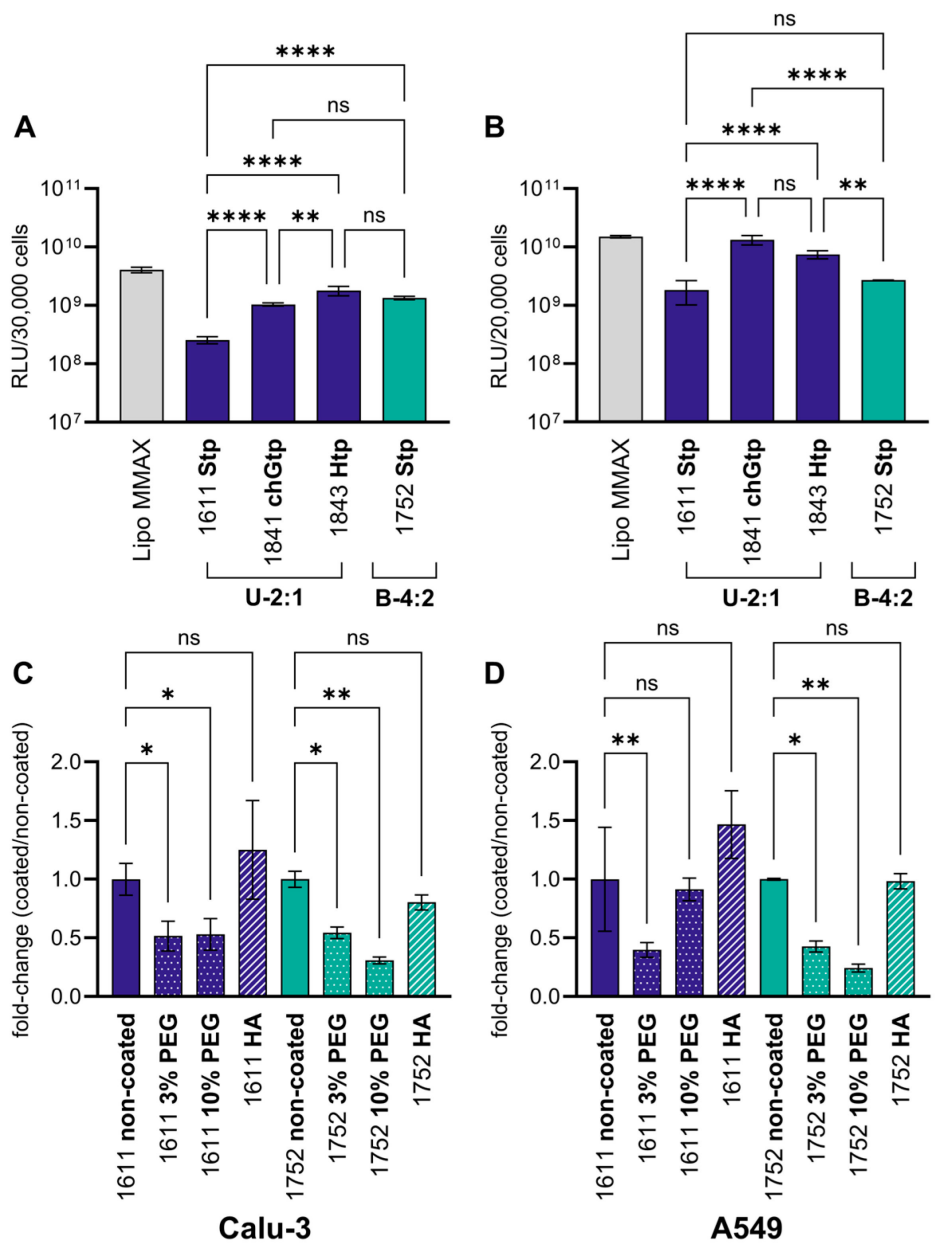
Luciferase expression under standard submerged culture conditions. Expression evaluated 24 h after transfection with indicated LAF-XP polyplexes at a dose of 12.5 ng mRNA-FLuc per well in (**A**,**C**) Calu-3 and (**B**,**D**) A549 cells. (**A**,**B**) mRNA expression of indicated LAF-XP polyplexes as relative light units (RLU). (**C**,**D**) Fold-change in luciferase expression of LAF-XP polyplexes with surface modification in relation to non-coated LAF-XP polyplexes. U-2:1 (N/P 18) and B-4:1 (N/P 24). Lipo MMAX is Lipofectamine MessengerMAX. (**A**,**B**) A lognormal ordinary one-way ANOVA with Tukey’s multiple comparisons test and (**C**,**D**) an ordinary one-way ANOVA with Šídák’s multiple comparisons test were performed. (ns *p* > 0.05, * *p* ≤ 0.05, ** *p* ≤ 0.01, **** *p* ≤ 0.0001). Blue: U-2:1, green: B-4:1, grey: Lipo MMAX, no pattern: non-coated, dots: PEGylated, slash stripes: HA-coated.

In vitro transfection efficiency was evaluated in human Calu-3 lung adenocarcinoma (Figure 3A,C) and human A549 lung carcinoma epithelial cells (Figure 3B,D) under standard submerged culture conditions in medium supplemented with 10% FBS. The different LAF-XP polyplexes with and without surface modifications were investigated. At 24 h after transfection with 12.5 ng mRNA per well, a luciferase expression assay revealed similar trends for both cell lines. In the case of Stp-based LAF-XPs, transfection efficiency was highest for bundle 1752, followed by small U-shape 1611 (12Oc/Stp 2:1). Lipophilic Stp-analogs Htp and chGtp improved the transfection efficiency of U-shapes up to 7-fold in Calu-3 and A549 cells. Bundle 1752 as well as U-2:1 based on Htp (1843) and chGtp (1841) achieved the highest transfection efficiencies (Figure 3A,B).

These data demonstrate a high translatability of transfection trends between adherent cell lines of different origins cultivated in submerged conditions such as murine and human cancer cells or dendritic cells; bundles and hydrophobized U-shapes show high endosomal release [37,38,39,76]. This suggests that the endosomal escape is also a major bottleneck in lung epithelial cells. PEG and HA surface-modified polyplexes of standard Stp-based U-shaped carrier 1611 and bundle 1752 were also evaluated under submerged conditions (Figure 3C,D). PEGylation with a molar ratio of 3% and 10% PEG-DMG of total carrier reduced the luciferase expression levels only moderately, as shown on the linear scale. HA coating applied to 1611 and 1752, in contrast, did not alter transfection efficiency. In sum, the choice of the specific LAF-XP in the mRNA nanoparticle core was the key factor influencing transfection efficiency in submerged culture.

**Figure 4:**
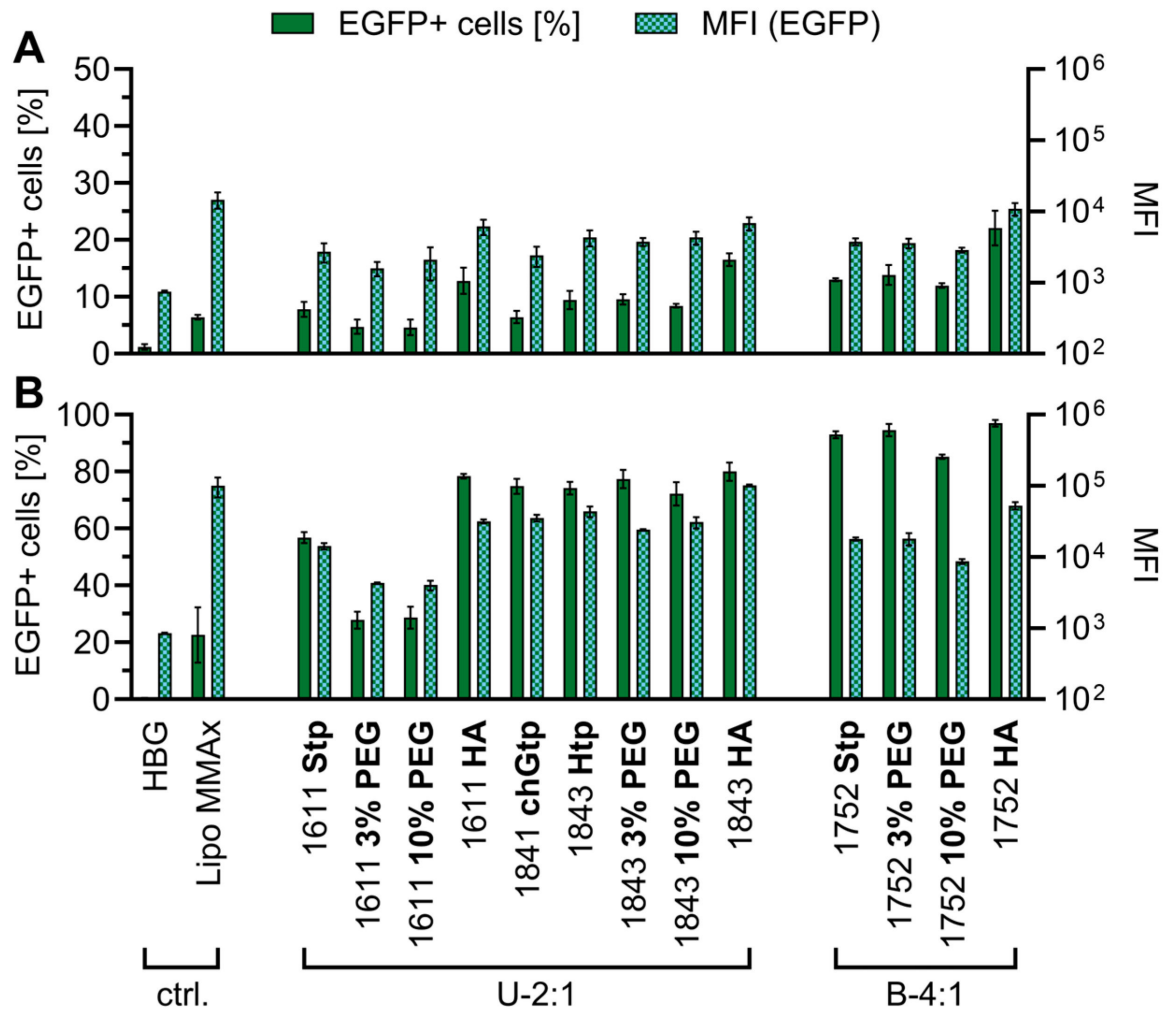
Flow cytometric evaluation of EGFP expression. Data presented as percentage of transfected cells and MFI of live cells in (**A**) Calu-3 cells and (**B**) A549 cells at 4 h after transfection with indicated LAF-XP polyplexes at a dose of 25 ng mRNA (2/8 mRNA-FLuc-Cy5/mRNA-EGFP) per well. U-2:1 (N/P 18), and B-4:1 (N/P 24).

For flow cytometric mRNA expression analysis, PEGylated and HA-coated best-performing U-shape 1843 (Htp) (DLS: Figure S1) were included. At the earlier point of 4 h after transfection with mRNA-EGFP, topology-dependent expression levels with B-4:1 outperforming U-2:1 were also found in both cell lines. Hydrophobic OAA modifications in U-shapes were especially beneficial in A549 cells. Interestingly, HA coating tended to be beneficial in both cell lines, whereas PEGylation was not (Figure 4). This was also true for Htp-based U-2:1 (1843).

Overall, EGFP expression levels after 4 h were in accordance with luciferase expression results at 24 h after transfection (Figure 3) but suggest a beneficial effect of HA coating at early time points. Enhanced uptake or a slight destabilization by negative charges, resulting in accelerated release of the cargo could be the reasons. PEGylation, in contrast, might hamper or delay cellular uptake and endosomal escape. A549 cells were easier to transfect than Calu-3 cells with 46–97% of EGFP-positive (EGFP+) cells already after 4 h compared to 2–22% in the case of Calu-3 cells.

An MTT assay at an mRNA dose of 50 ng per well revealed a relative metabolic activity over 90% for Calu-3 cells and over 70% in case of A549 cells for all tested formulations, indicating good biocompatibility in both cell lines (Figure S2).

### 3.5. Transfection Under Air–Liquid Interface Conditions

**Figure 5:**
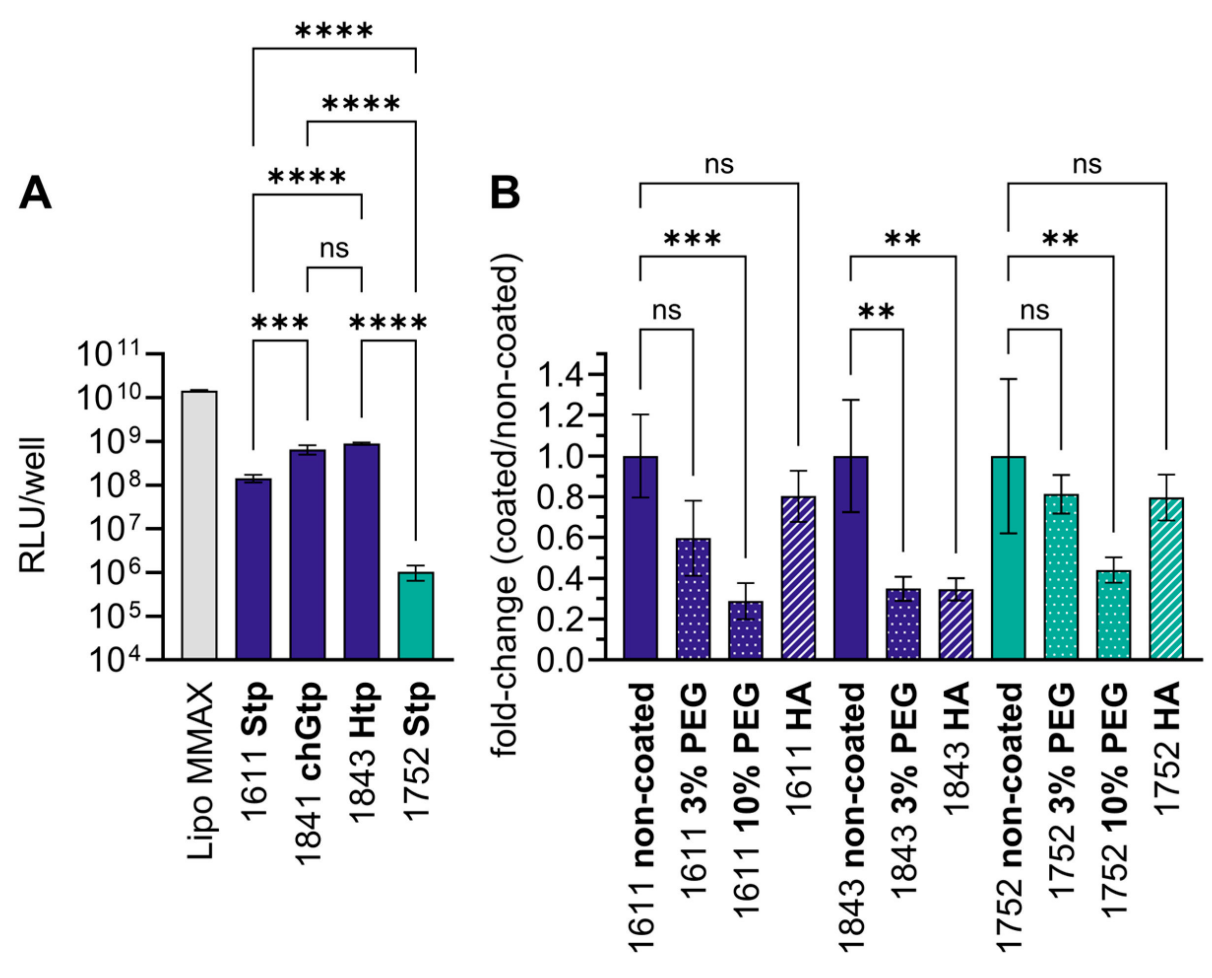
Luciferase expression assay in Calu-3 cells cultivated as air–liquid interface (ALI) culture. Read-out 24 h after transfection with a dose of 300 ng mRNA-FLuc per well. (**A**) Luciferase expression of indicated non-coated LAF-XP polyplexes presented as relative light units (RLU) per well. (**B**) Alteration of the luciferase expression of indicated LAF-XP polyplexes with surface modification (PEG or HA) in relation to non-coated LAF-XP polyplexes represented as fold-change in RLU/well of coated to the respective non-coated LAF-XP polyplexes. U-2:1 (N/P 18), and B-4:1 (N/P 24). (**A**) A lognormal ordinary one-way ANOVA with Tukey’s multiple comparisons test and (**B**) an ordinary one-way ANOVA with Šídák’s multiple comparisons test were performed (ns *p* > 0.05, ** *p* ≤ 0.01, *** *p* ≤ 0.001, **** *p* ≤ 0.0001). Blue: U-2:1, green: B-4:1, grey: Lipo MMAX, no pattern: non-coated, dots: PEGylated, slash stripes: HA-coated.

A common in vitro model mimicking the special conditions of topical pulmonary drug delivery are air–liquid interface (ALI) cell culture models with mucus-secreting cells. Among them, Calu-3 cells represent a common ALI model for lung epithelium as they grow in polarized, columnar monolayers rich in tight junctions, can secrete mucus, and express the CFTR gene [77,78,79,80,81]. Therefore, the transfection efficiency was evaluated in Calu-3 cells cultured under mucus-producing ALI conditions at least for one week before transfection [50]. LAF-XP polyplexes were formulated as described above with mRNA diluted in HBG with 10% (*w*/*v*) glucose (HB2xG) to ensure a final isosmotic formulation after mixing with the aqueous carrier dilution. A dose of 300 ng mRNA-FLuc was applied to the mucus-covered surface of the Calu-3 cells. Luciferase expression was measured 24 h after transfection (Figure 5). All U-shapes clearly outperformed the bundle topology. Carrier 1611 showed a 139-fold increase in relative light units (RLU) compared to 1752, and 1843 showed 855-fold higher expression than the bundle (Figure 5A). In contrast, HA coating or PEGylation induced only minor reductions in factors ≥0.3 or did not lead to significant changes at all for both topologies (Figure 5B). This was especially interesting as HA-coating substantially altered the physicochemical characteristics (i.e., larger size, negative zeta potential) (Figure 1). Improvements were observed for U-shapes with hydrophobized OAAs chGtp and Htp. Htp achieved a 6-fold increase in RLU values compared to Stp for U-2:1 and carrier 1843 was identified as best overall performer.

**Figure 6:**
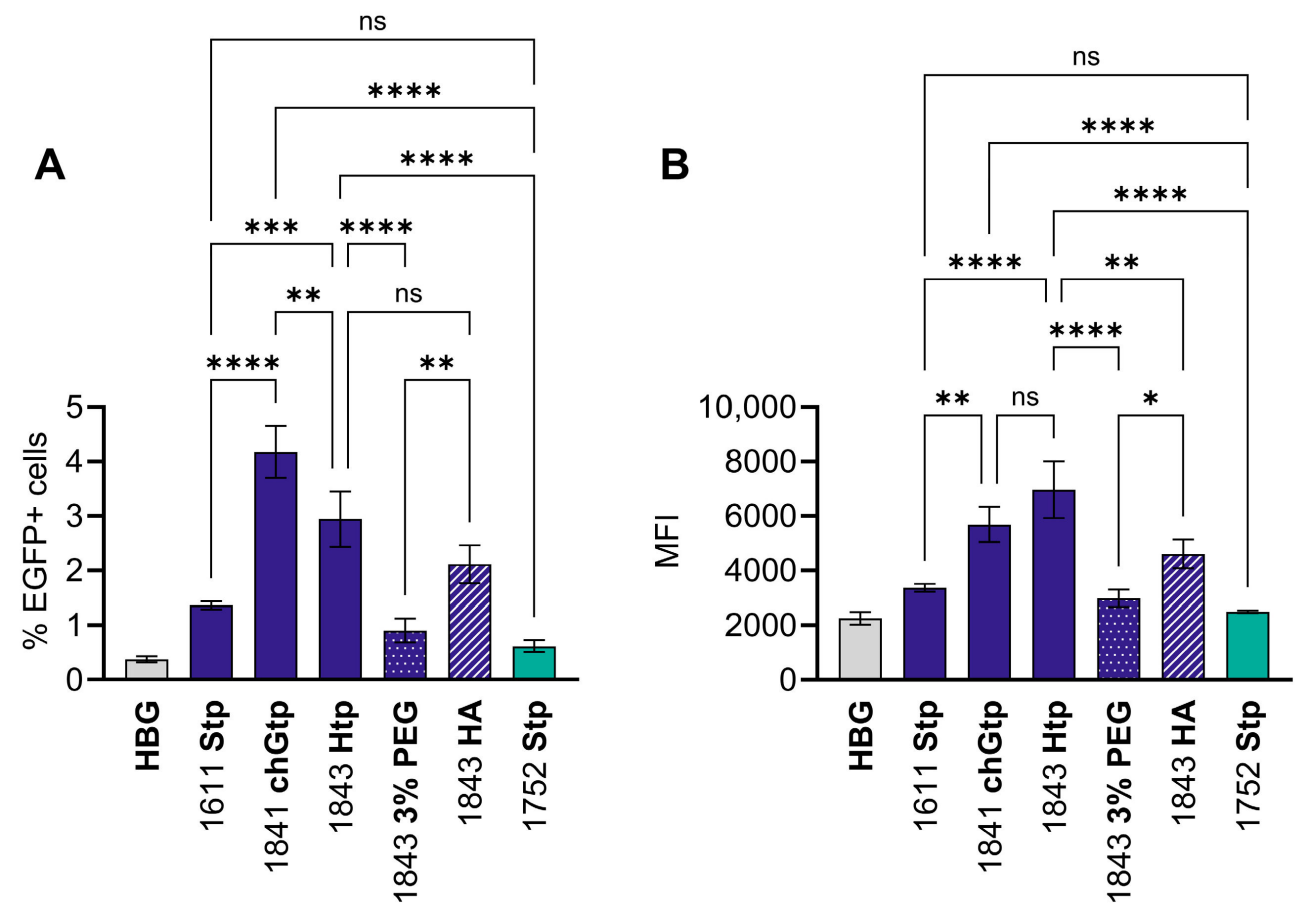
Flow cytometry analysis at 24 h after transfection of Calu-3 cells cultivated as air–liquid interface culture transfected with a dose of 600 ng mRNA-EGFP per well. U-2:1 (N/P 18), and B-4:1 (N/P 24). (**A**) Transfection efficiency depicted as percentage of EGFP expressing (EGFP+) cells. (**B**) Transfection efficiency complementary evaluated as intensity of EGFP expression presented as mean fluorescence intensity (MFI) of EGFP in the live cell population. An ordinary one-way ANOVA with Tukey’s multiple comparisons test was performed and relevant comparisons are depicted in the graph. (ns *p* > 0.05, * *p* ≤ 0.05, ** *p* ≤ 0.01, *** *p* ≤ 0.001, **** *p* ≤ 0.0001). Grey: negative control, blue: U-2:1, green: B-4:1, no pattern: non-coated, dots: PEGylated, slash stripes: HA-coated.

Complementary, transfection efficiency in ALI-cultivated Calu-3 cells was analyzed with selected mRNA-EGFP containing LAF-XP polyplexes (Figure 6). The Htp- and chGtp-containing U-shape carriers transfected the highest number of cells (3–4%), whereas the bundle structures reached <1% (Figure 6A). The U-shape carrier 1841 was most potent with ~4% EGFP+ cells. PEGylation of 1843 reduced the percentage whereas HA coating, once again, did not significantly alter the transfection efficiency. The mean fluorescence intensity (MFI) of the live cell population (Figure 6B) supported the trends observed in the luciferase expression assay (Figure 5) and confirmed U-shape LAF-XP polyplexes with hydrophobized OAA domain and without surface modification as most promising in ALI Calu-3 culture. Generally, only a small cell population was transfected, raising interest in cellular uptake and association which will be discussed later in this manuscript.

Notably, the expression levels in mucus-covered Calu-3 cells cultured under ALI conditions showed different trends for beneficial LAF-XP topologies compared to submerged culture. In the ALI model, U-shapes clearly outperformed bundle-based LAF-XP polyplexes with an up to 855-fold (1843 compared to 1752) increase in luciferase expression (Figure 5). Bundles are more lipophilic due to their relatively higher content of LAFs (LAF/OAA 4:1). This could cause interactions with hydrophobic domains of the mucins which could be detrimental for mucus penetration [6,30,32]. A different protein corona after exposure to lung secretion, which alters protein composition and abundance compared to serum, could alter the biological identity of the particle and influence uptake and/or intracellular trafficking. [82,83,84,85] Additionally, bundles exhibit a lower colloidal stability [37,42] which might be detrimental in the presence of mucus. They also tend to have higher cytotoxicity compared to U-shapes [37,76], although an MTT assay (Figure S2) indicated high biocompatibility on Calu-3 cells.

Unexpectedly, neither anionic surface coating with HA nor colloidal stabilization [42] and hydrophilization by PEGylation enhanced expression levels in ALI-cultured cells (Figure 5 and Figure 6). Distinct properties of the core polyplex such as lipid phase formation [40] or endosomal escape behavior [37,38,39] might be more decisive. Specifically, U-shapes form classical lamellar lipid phases, as analyzed by small angle X-ray scattering (SAXS), and require endosomal acidification for transfection efficiency. In contrast, bundles form fusogenic, bicontinuous cubic lipid phases and are barely dependent on endosomal acidification [40]. For U-shapes with hydrophobized OAAs, a reduced dependence on endosomal acidification was also found [38].

The results might also reflect competing effects. In the case of HA coating, a positive effect of a hydrophilic particle surface might be mitigated by electrostatic repulsion between negatively charged mucins and particles with clearly negative zeta potential (<15 mV). Bandi et al. and Lieleg et al. reported a benefit of neutral over negatively charged polystyrene nanoparticles for mucus penetration [26,86]. The larger particle size compared to non-coated LAF-XP polyplexes might hamper mucin mesh penetration. The reported pore size of the mucus mesh varies between 100 and 500 nm [6,23,27,30,87,88], while also larger low-viscosity channels are reported [30,89]. However, small nanoparticles are often reported to be beneficial to traverse the mucus barrier [6,26,27,90]. Addition of a second negatively charged polyanion besides mRNA into the delivery system could also decrease particle stability, although gel shift assays (Figure 1E,F) did not indicate mRNA release.

The reduced efficiency of PEGylated particles might reflect the “PEG-dilemma”, describing the tradeoff between efficient shielding and stabilization at the expense of reduced cellular uptake and diminished endosomal escape due to lower membrane interaction [55,56]. Positive effects of PEGylation in topical pulmonary delivery are traced to reduced mucoadhesion and enhanced mucus penetration and referred to as gold standard for overcoming the mucus barrier [21,24,29,30,31,32]. Yet, optimization of the PEG-chain length and density to the respective nanoparticle can be crucial to exploit the full potential of PEGylation for mucus penetration and simultaneously avoid the drawbacks in reduced transfection [21,87,88,91,92,93]. For example, Grun et al. reported an optimum of 5%- PEGylated poly(amine-co-ester) for local pulmonary mRNA delivery and a negative effect of higher PEGylation degrees [28]. Conte et al. even showed that PEGylation did not support siRNA lipid-polymer hybrid nanoparticles in traversing complex, pathologic mucus samples [77] and Boylan et al. found insufficient diffusion of PEGylated polylysine-based DNA polyplexes through viscous cystic fibrosis sputum, although gene expression had been observed upon inhalative lung administration in healthy BALB/c mice [93].

While the most effective LAF-XP topologies differed between submerged and ALI cell culture, the trends related to hydrophobic OAA modifications of carriers remained largely unchanged (Figure 3A and Figure 5A). As described by Burghardt et al., Htp and chGtp did not strongly alter physicochemical particle properties compared to Stp but mostly changed the endosomal escape activity, as shown by enhanced calcein release, and reduced impact of the endosomal vATPase inhibitor bafilomycin A1 [38]. Obviously, this beneficial intracellular fate was maintained in ALI culture. Overall, the transfection efficiency was predominantly governed by carrier topology.

### 3.6. Transfection Efficiency with and Without Serum

Standard submerged cell culture and ALI culture strongly differ in exposure to serum proteins, just as under differing in vivo (intravenous versus intratracheal) conditions. To further explore the influence of serum on the transfection efficiency, submerged Calu-3 cells were transfected, as standard in the presence of 10% FBS, as well as in serum-free medium. To avoid reduced cellular health due to the lack of serum, the transfection medium was replaced 4 h after transfection with medium supplemented with 10% FBS in all wells. Luciferase expression was measured 24 h after transfection. The results showed that U-shapes clearly benefitted from serum-free transfection conditions, whereas bundles did not show differences (Figure S3). The efficiency of U-shape carriers surpassed bundles in a serum-free transfection. Surface modifications did not alter this trend. These findings confirmed the structural topology of the carriers as critical parameter for transfection efficiency of LAF-XP polyplexes under different transfection conditions. The benefit of the U-shape topology was demonstrated in serum-free transfection conditions, not only in ALI culture but also in submerged culture. As colloidal stabilization by PEGylation [42] did not alter the relations, aggregation could be excluded as driving factor.

### 3.7. Cellular Association and Uptake

Due to the mucus barrier and the low endocytic activity of lung epithelium, achieving cell association and cellular uptake is one of the key bottlenecks in pulmonary delivery [5,13,21,24,94,95,96].

Under standard submerged conditions, uptake was observed in ~20–30% of Calu-3 cells 4 h after transfection (Figure S4A). LAF-XP polyplexes without surface modification did not significantly differ, whereas an increased percentage of Cy5-positive cells with HA-coated LAF-XP polyplexes was observed for 1611 and 1752. PEGylation was not disadvantageous. In A549 cells, uptake was detected in >90% of cells irrespective of the LAF-XP and surface modification (Figure S4B). HA coating tended to increase the amount of uptake per cell as represented by the MFI of Cy5-positive (Cy5+) cells, whereas PEGylation showed reductions in MFI. The slightly increased uptake of HA-coated particles was observed for both CD44+ A549 and CD44-Calu-3 cells [64,97]. LipoMMAX was taken up by <25% of A549 cells and only 9% of Calu-3 cells with a high MFI, suggesting an inhomogeneous uptake pattern.

In Calu-3 cells cultured under ALI conditions, cell association and uptake were evaluated at 6 h after transfection (Figure S4C,D). LAF-XP polyplexes without surface modification reached 29–37% of cells without significant differences between the groups (Figure S4C). The highest percentage of Cy5+ cells was achieved with PEGylated 1843 (51%). MFIs of Cy5+ cells indicating the uptake per cell did not strongly differ (Figure S4D).

In summary, a discrepancy between cell association and uptake on the one hand, and expression on the other hand was observed, especially in Calu-3 ALI culture. In the latter, the percentage of Cy5+ cells (30–51%) (Figure S4C) exceeded the percentage of EGFP+ cells (Figure 6A) by more than 10-fold. The slight benefit of hydrophilic surface modifications for the uptake was not reflected in enhanced expression levels. Differentiated ALI-cultured epithelial cells are hard to transfect, especially from the apical side, not only because of the mucus barrier but also due to low endocytosis activity [5,13,94,95,96,98]. They grow as dense monolayers with high transepithelial electrical resistance (TEER) values [78]. Therefore, high apical cell association but low internalization might be one reason for this discrepancy. Additionally, in submerged culture of Calu-3 cells, only minor differences between uptake of the different LAF-XP polyplexes were observed while early EGFP expression varied (Figure 4A). This suggests that besides accessing the cell, the intracellular fate of the particles is critical in this cell line.

### 3.8. In Vivo Intratracheal Administration

For in vivo evaluation of local pulmonary administration, Stp-based LAF-XPs of U-shape (1611) and bundle (1752) topology were selected, as well as the U-shapes with hydrophobized OAAs (1841 chGtp and 1843 Htp). Additionally, 3% PEG-DMG and HA coating were applied to ALI culture best performer 1843 to investigate the influence of surface modification in vivo. DLS measurements at in vivo mRNA concentration (60 µg/mL) showed particle sizes ranging from 99 to 156 nm, zeta potentials of >20 mV for LAF-XP non-coated polyplexes, −19 mV for HA-coated, and 16 mV for PEGylated 1843 (Table S2), in line (Figure 1 and Figure S1).

**Figure 7:**
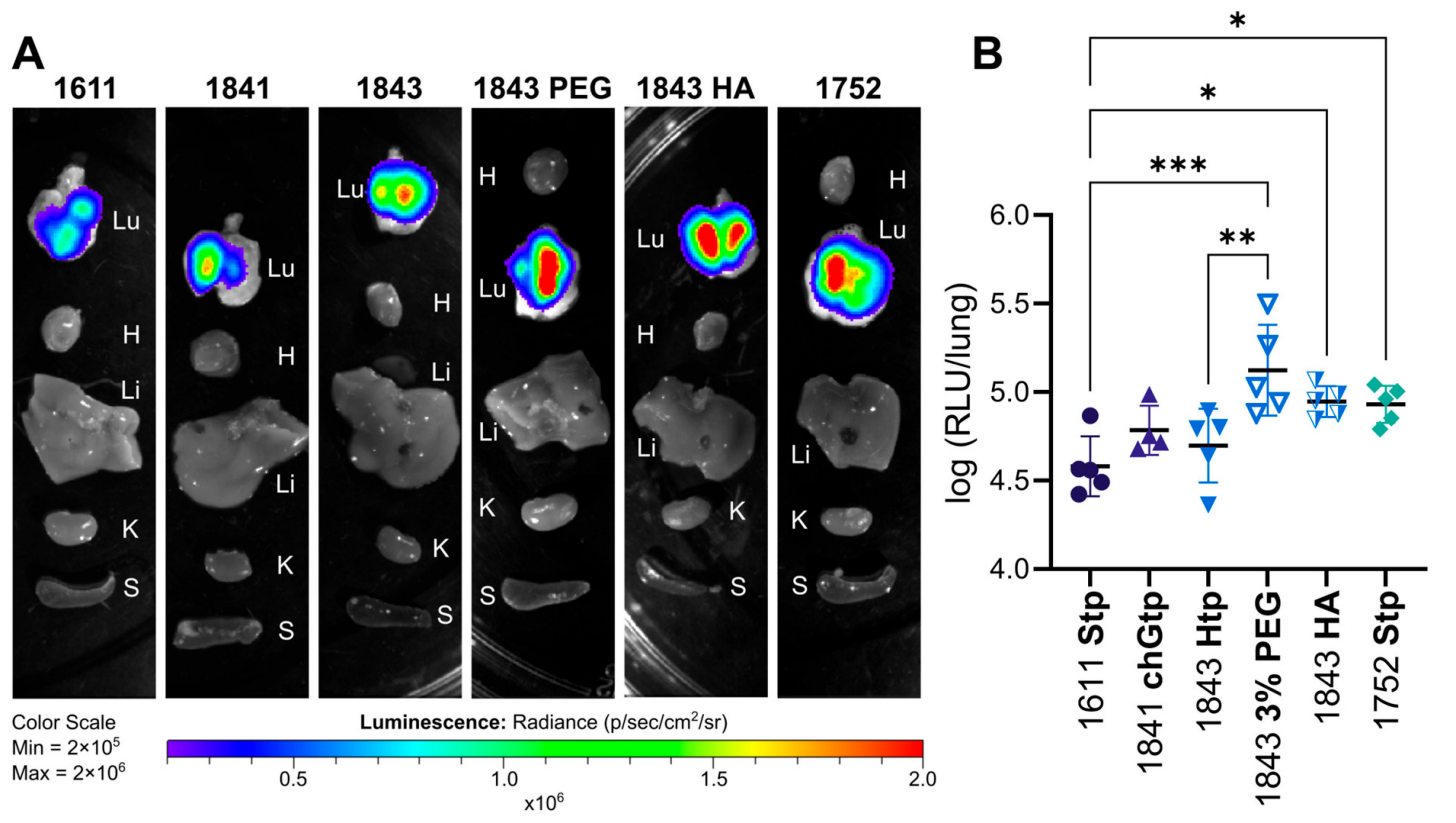
Intratracheal in vivo administration. Indicated LAF-XP polyplexes were administered intratracheally to BALB/c mice (*n* = 5; 1841 *n* = 4) at a dose of 3 µg of mRNA-FLuc per mouse. Necropsy and organ explantation were performed 6 h after administration. (**A**) IVIS imaging after intraperitoneal administration of D-luciferin. Representative organ images of one mouse per group. (**B**) Luciferase expression in lung homogenates. Ordinary one-way ANOVA with Tukey’s multiple comparisons test for log(RLU/g organ) values. All significant group comparisons are shown in the graph. * *p* ≤ 0.05; ** *p* ≤ 0.01; *** *p* ≤ 0.001.

Topical pulmonary transfection efficiency in vivo was investigated after intratracheal instillation. This approach enables direct comparison of the transfection performance without impairment by additional delivery hurdles. Alternative application routes like nebulization can impose additional challenges such as shear force affecting particle stability [99,100], the need for additional excipients and buffer optimization [13,101], and device-dependent variability [101,102]. Formulation development for nebulized delivery would be a valuable follow-up after identifying efficient delivery agents. Thus, a dose of 3 µg mRNA-FLuc per mouse was administered by intratracheal instillation in a total volume of 50 µL and read-out was performed 6 h after administration with an in vivo optical imaging system (IVIS, Xenogen, Perkin Elmer, Shelton, CT, USA) (Figure 7A) and a subsequent ex vivo luciferase expression assay of lung homogenates (Figure 7B). The data showed local mRNA expression in the lungs without detectable signals in other organs (Figure 7A). Quantitative analysis of homogenized lung tissue did not reveal significant differences between U-shapes without surface modification (Figure 7B). Remarkably, bundle 1752, which induced the lowest expression levels in ALI culture (Figure 5), significantly outperformed the Stp-based U-shape 1611 in vivo. Highest expression levels were obtained with Htp-containing U-shape carrier 1843 modified with 3% PEG-DMG, followed by HA-coated 1843 (Figure 7B). Both groups significantly exceeded expression levels of the standard Stp-based U-shape 1611. HA coating doubled the expression level, whereas PEGylation even enhanced luciferase expression 4-fold. PEGylated 1843 also significantly outperformed non-coated 1843, whereas HA-coating did not (Figure 7B).

The data show an in vitro–in vivo discrepancy which could have several reasons. As Calu-3 ALI culture can exhibit TEER values even higher than the natural human airway epithelium [103], the model might be more pessimistic than results obtained in in vivo studies. The secreted mucus of Calu-3 cells and other ALI cultures also shows differences from native human airway mucus such as in the abundance and composition of secreted types of mucins [104], the compositional complexity such as the content of other proteins, DNA or immune cells, or rheological properties [105]. Moreover, in vitro models do not exhibit mucociliary clearance, thereby not reflecting one major pitfall in pulmonary delivery. In addition, the bronchial surface does not only consist of epithelial cells but also other cell types, and cell morphology such as the occurrence of cilia might differ, making mono-cellular ALI culture a simplified model [78,79,80,98].

## 4. Conclusions

In summary, the study points to a trade-off between distinct mitigation strategies aimed at overcoming multiple delivery barriers in pulmonary mRNA delivery. The sweet spot between particle size, colloidal stability and surface properties, as well as the particle’s core stability and intracellular fate, needs to be found. The optimal balance between mucus penetration, cellular uptake, and efficient intracellular fate depends on the cellular environment.

Transfections in lung epithelial cells in submerged conditions with and without serum supplementation, as well as under ALI conditions, revealed different best performers. Small, stable U-shape carriers proved particularly effective in serum-free and ALI conditions, whereas the presence of serum favored bundle carriers. Hydrophilic surface modification by PEG or HA altering physicochemical particle properties (size, zeta potential) did not have similar strong impact in vitro as compared to the carrier architecture. In contrast, even small modifications within the polar OAA domain of the XPs, recently shown to enhance the endosomal escape, showed their beneficial effect in all tested transfection models. Under submerged conditions, Htp (1843) and chGtp (1841) mediated a 7-fold enhanced luciferase expression compared to the initial Stp-based U-shape 1611 in Calu-3 and A549 cells, respectively. In ALI conditions, 1843 induced a 6-fold increase in luciferase expression compared to 1611 and an 855-fold increase compared to bundle 1752. An in vivo experiment with intratracheal administration into mice highlighted the need for careful investigation of the transfection conditions. The use of LAF-XPs with efficient endosomal escape properties and eventually the addition of hydrophilic surface modifications achieved local pulmonary mRNA expression. An LAF-XP polyplex based on 1843 modified with 3% PEG-DMG achieved 4-fold higher pulmonary expression levels in vivo compared to the initial 1611 carrier.

The results show that local pulmonary delivery is a highly delicate administration route, where particles need to be fine-tuned and in vitro results cannot always be translated. The study reports the first application of LAF-XP polyplexes for local pulmonary mRNA delivery and the implementation of HA coating to this carrier system. It contributes to extending the field of local pulmonary delivery systems beyond LNPs by giving insight into optimization strategies for polyplex mediated delivery.

## Data Availability

The original contributions presented in this study are included in the article/Supplementary Materials. Further inquiries can be directed to the corresponding author.

## Supplementary Materials

The following supporting information can be downloaded at https://www.mdpi.com/article/10.3390/polym18111368/s1, Table S1: Diffusion of non-coated and HA-coated 1611 LAF-XP polyplexes in HBG; Figure S1: DLS/ELS of 1843 with surface modification; Figure S2: Relative metabolic activity evaluated via MTT assay; Figure S3: Submerged transfection in the presence and absence of serum; Figure S4: Cellular uptake and association; Table S2: Size and zeta potential of LAF-XP polyplexes for in vivo administration.

## Abbreviations

The following abbreviations are used in this manuscript:

12Bu	4-(didodecylamino)butyric acid
12Oc	8-(didodecylamino)octanoic acid
ACF	autocorrelation function
ALI	air–liquid interface
chGtp	3-(cyclohexyl)glutaroyl tetraethylene pentamine
CCF	cross-correlation function
Cy5+	Cy5-positive
DMEM	Dulbecco’s Modified Eagle’s Medium
EGFP	enhanced green fluorescent protein
EGFP+	EGFP-positive
FBS	fetal bovine serum
FCCS	fluorescence cross-correlation spectroscopy
FCS	fluorescence correlation spectroscopy
FLuc	firefly luciferase
HA	hyaluronic acid
HBG	HEPES buffered glucose
Htp	hexahydrophthaloyl tetraethylene pentamine
LAF	lipoamino fatty acid
Lipo MMAX	Lipofectamine MessengerMAX Reagent
LNP	lipid nanoparticle
MEM	Minimum Essential Medium Eagle
MFI	mean fluorescence intensity
mRNA	messenger ribonucleic acid
OAA	oligoamino acid
PBS	phosphate-buffered saline
PEG	poly(ethylene glycol)
PEG-DMG	1,2-dimyristoyl-rac-glycero-3-methoxypolyethylene glycol-2000
PEI	polyethylenimine
SAXS	small-angle X-ray scattering
Stp	succinoyl tetraethylene pentamine
TEER	transepithelial electrical resistance
XP	xenopeptide
