# Supplementary material for: mRNA Delivery by Lipoamino Fatty Acid–Peptide Polyplexes in Different Lung Cell Models and Lungs
Source: Polymers (Basel). 2026 May 31;18(11):1368. doi: 10.3390/polym18111368 (PMC13259057; doi:10.3390/polym18111368)
Supplement: Supplementary file 1 [file polymers-18-01368-s001.zip › polymers-4322165-supplementary.pdf]

Supporting Information

mRNA Delivery by Lipoamino Fatty Acid-Peptide Polyplexes in Different Lung Cell Models and Lungs

Sophie Thalmayr †, 1, 2, 3, Joschka Müller †, 3, 4, Vivien Polewka 3, 5, Irene Gialdini 2, 6, Anny Nguyen 3, 4, Christian Dohmen 3, 5, Don C. Lamb 2, 6, Olivia M. Merkel 2, 3, 4, Ernst Wagner *, 1, 2, 3

1 Pharmaceutical Biotechnology, Department of Pharmacy, Ludwig-Maximilians-Universität München, Munich, 81377 Munich, Germany; sophie.thalmayr@cup.uni-muenchen.de (S.T.)

2 Center for NanoScience (CeNS), Ludwig-Maximilians-Universität München, 80799 Munich, Germany.

3 CNATM - Cluster for Nucleic Acid Therapeutics Munich, 81377 Munich, Germany.

4 Pharmaceutical Technology and Biopharmaceutics, Department of Pharmacy, Ludwig-Maximilians-Universität München, 81377 Munich, Germany; joschka.mueller@cup.uni-muenchen.de (J.M.); anny.nguyen@cup.uni-muenchen.de (A.N.); olivia.merkel@lmu.de (O.M.M.)

5 Ethris GmbH, Planegg, 82152, Germany; dohmen@ethris.com (C.D.); polewka.viven@mh-hannover.de (V.P.)

6 Department of Chemistry, Ludwig-Maximilians-Universität München, 81377 Munich, Germany; irene.gialdini@cup.lmu.de (I.G.); d.lamb@lmu.de (D.C.L.)

† These authors contributed equally to this work.

* Correspondence: ernst.wagner@cup.uni-muenchen.de (E.W.)

1. Supporting Figures and Tables

Table S1 Diffusion of non-coated and HA-coated 1611 LAF-XP polyplexes in HBG. FCS values obtained from fitting the ACFs.

	Mean f_1 ± SD	Mean D_1 (μm²/s) ± SD	Mean f_2 ± SD	Mean D_2 (μm²/s) ± SD
**Controls**	**mRNA/HA species**		**Free dye species**	
mRNA-ATTO565	0.97 ± 0.05	10.2 ± 0.6	0.03 ± 0.05	386*
HA-ATTO643	0.79 ± 0.04	17.2 ± 1.7	0.21 ± 0.04	87 ± 19**
**Polyplexes**	**Polyplex species**		**Free mRNA/HA species**	
Non-coated NPs, yellow ACF	0.26 ± 0.02	1.4 ± 0.3	0.74 ± 0.02	10.2 (fixed as mRNA)
Non-coated NPs, red ACF	1	0.15 ± 0.21***	-	-
Coated NPs, yellow ACF	0.58 ± 0.03	1.75 ± 0.40	0.42 ± 0.03	10.2 (fixed as mRNA)
Coated NPs, red ACF	0.97 ± 0.04	1.97 ± 0.50	0.03 ± 0.04	17.2 (fixed as HA)

The autocorrelation functions (ACFs) of the yellow channel (560 nm excitation, ATTO565 labeled species) and red channels (635 nm excitation, ATTO643 labeled species) were analyzed using a one or two-component diffusion model. The parameters f1 and f2 represent the relative fraction of species 1 and 2, obtained from fitting the relative amplitudes within the ACF. It should be noted, since an accurate quantification of the number of particles cannot be performed, f1 and f2 are approximations. The identity of each species is indicated in the table head, unless otherwise specified. The reported values represent the mean ± SD of 2 independent FCS experiments, as indicated in the last column.

* The second species is only present in one replicate and is attributed to free dye (ATTO565) cleaved from the mRNA.

**The second species show a slower diffusion than expected for free dye might represent dye association with HA fragments.

*** Artifact value obtained from spurious fit of the correlation.

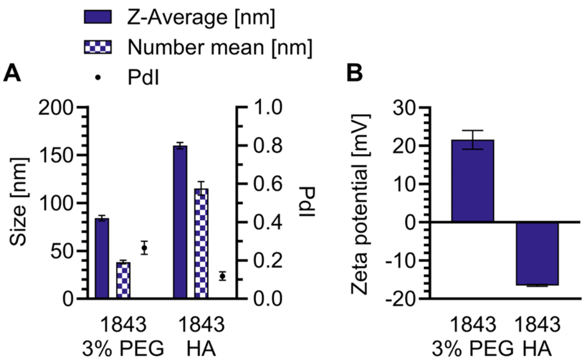

Figure S1 DLS/ELS of 1843 with surface modification. A) Size, PdI and B) zeta potential of 1843 polyplexes with 3% molar ratio of PG-DMG or HA coating with 3 HA/OAA. For particle formation, mRNA was diluted HB2xG.

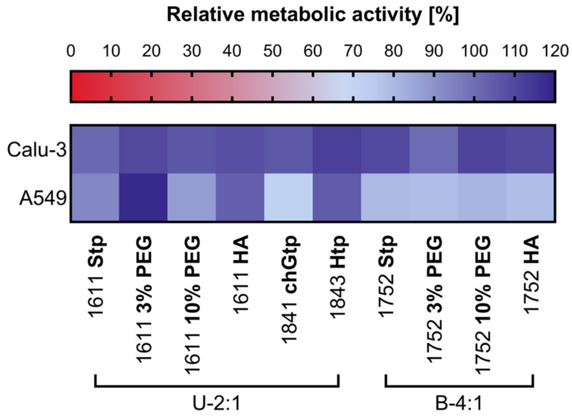

Figure S2 Relative metabolic activity evaluated via MTT assay. Calu-3 cells and A549 cells were transfected with indicated LAF-XP polyplexes at a dose of 50 ng mRNA-FLuc per well and an MTT assay was performed at 24 h after transfection. Control wells with HBG treatment were used as reference for 100% metabolic activity. U-2:1 (N/ 18), and B-4:1 (N/P 24)

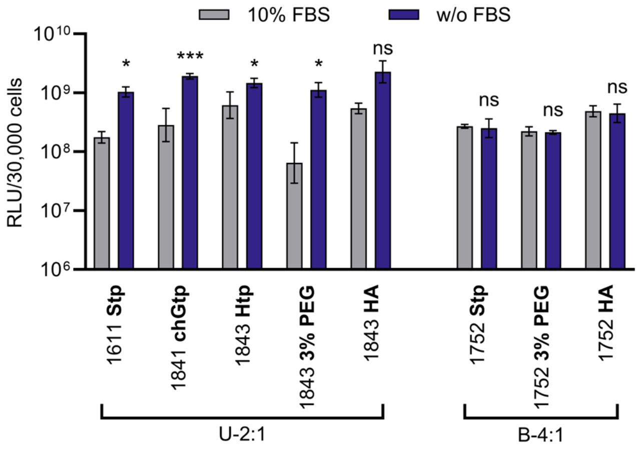

Figure S3 Submerged transfection in the presence and absence of serum. Luciferase expression assay 24 h after transfection of Calu-3 cells with a dose of 12.5 ng mRNA-FLuc per well in either serum-free medium or medium supplemented with 10% FBS. Medium change to serum-supplemented medium at 4h after transfection. U-2:1

(N/P 18) and B-4:1 (N/P 24). Unpaired student's t-tests with Welch's correction were performed to compare the transfection conditions for each carrier (ns P > 0.05, * P ≤ 0.05, ** P ≤ 0.01, *** P ≤ 0.001, **** P ≤ 0.0001).

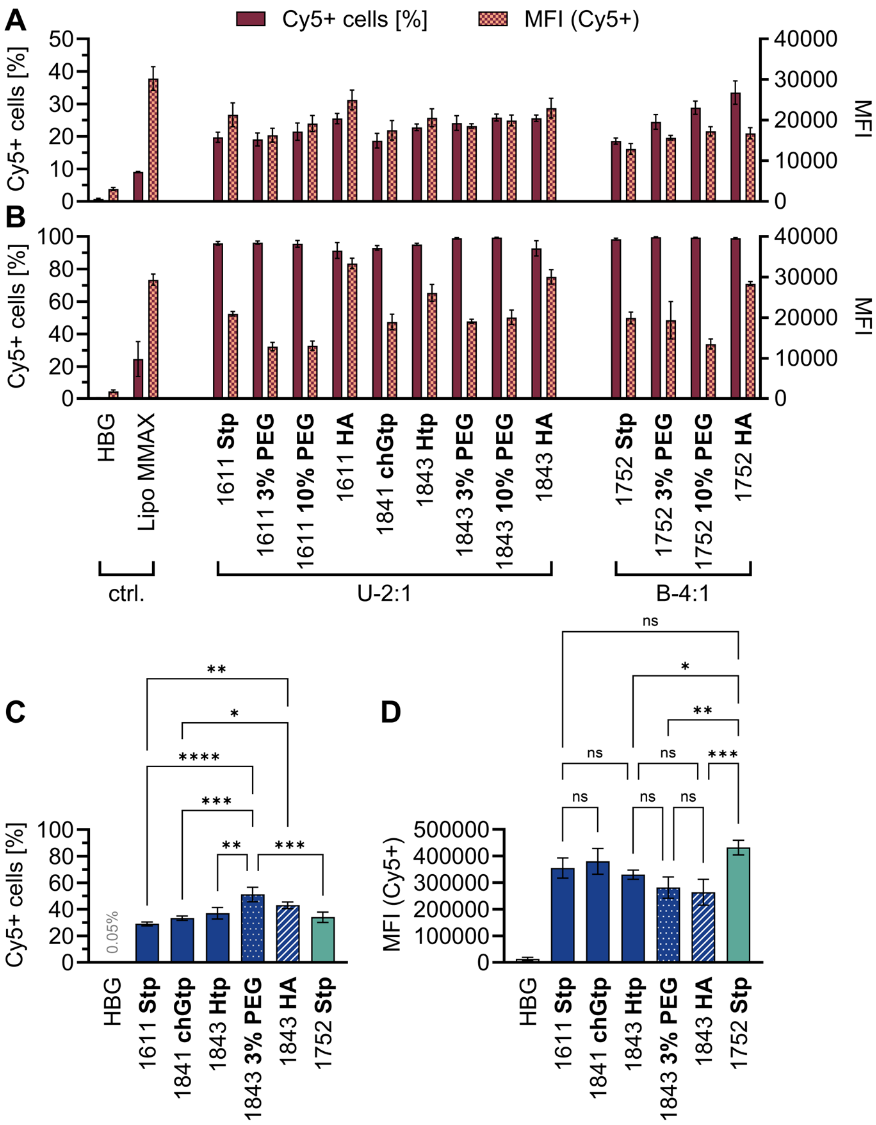

Figure S4 Cellular uptake and association. A, B) Flow cytometry analysis of cellular uptake of Cy5-labeled LAF-XP polyplexes in A) Calu-3 cells and B) A549 cells at 4 h after transfection under submerged conditions with indicated LAF-XP polyplexes at a dose of 25 ng mRNA (2/8 mRNA-FLuc-Cy5/mRNA-EGFP) per well. C, D) Flow cytometry analysis of cellular uptake and association at 6 h after transfection of Calu-3 cells cultivated in air-liquid-interface culture transfected with a dose of 600 ng mRNA (20% (w/w) Cy5-mRNA-FLuc, 80% (w/w) mRNA-EGFP) per well. C) Percentage of Cy5-positive (Cy5+) cells. D) MFI of Cy5 in the Cy5+ cell population. U- 2:1 (N/P 18) and B-4:1 (N/P 24). An ordinary one-way ANOVA with Tukey's multiple comparisons test was performed and relevant comparisons are depicted in the graph. (ns P > 0.05, * P ≤ 0.05, ** P ≤ 0.01, *** P ≤ 0.001, **** P ≤ 0.0001).

Table S2 Size and zeta potential of LAF-XP polyplexes for *in vivo* administration. Particle formation at a concentration of 0.06 mg/mL mRNA-FLuc (synthesized by Ethris, Planegg, Germany).

Topology	ID	OAA	Surface modification	Z-Average [nm]		Number mean [nm]		PdI		Zeta potential [mV]	
				mean	SD	mean	SD	mean	SD	mean	SD
U-2:1	1611	Stp		109.1	2.2	48.3	8.3	0.230	0.014	29.9	1.2
	1841	chGtp		98.7	1.5	37.2	5.7	0.208	0.011	28.8	0.6
	1843	Htp		114.0	0.6	33.3	14.7	0.209	0.019	29.2	1.5
	1843	Htp	3% PEG	100.5	1.5	48.9	21.9	0.195	0.020	16.0	0.5
	1843	Htp	HA	155.8	1.1	102.8	7.4	0.137	0.027	-19.2	0.6
B-4:1	1752	Stp		116.2	2.3	52.3	8.2	0.229	0.007	22.7	0.9

2. Supporting Methods

Relative metabolic activity (MTT assay).

Transfections were performed as described for the luciferase expression assay und submerged conditions with the following minor changes: cell culture medium was replaced with 96 µL fresh medium per well and relative metabolic activity was assessed at a dose of 50 ng mRNA (*i.e.*, 4 µL LAF-XP polyplex solution). At 24 h after transfection, 10 µL of MTT (5 mg/mL) was added to each well and cells were incubated for another 2 h at 37 °C. Subsequently, the medium was removed, and the plates were stored at -80 °C at least overnight. For measurement, the purple formazan was dissolved in 100 µL DMSO (30 min, 37 °C, constant shaking) and absorbance was measured at $\lambda = 590$ nm with background correction at $\lambda = 630$ nm by a Tecan Spark microplate reader (Tecan, Männedorf, Switzerland). Experiments were carried out in triplicates. Relative metabolic activity in relation to HBG-treated control cells was calculated by the equation:

$$A_{sample} \div A_{control} \times 100\% = relative\ metabolic\ activity$$
